# A fibroblast-dependent TGF-β1/sFRP2 noncanonical Wnt signaling axis promotes epithelial metaplasia in idiopathic pulmonary fibrosis

**DOI:** 10.1172/JCI174598

**Published:** 2024-07-09

**Authors:** Max L. Cohen, Alexis N. Brumwell, Tsung Che Ho, Kiana Garakani, Genevieve Montas, Darren Leong, Vivianne W. Ding, Jeffrey A. Golden, Binh N. Trinh, David M. Jablons, Michael A. Matthay, Kirk D. Jones, Paul J. Wolters, Ying Wei, Harold A. Chapman, Claude Jourdan Le Saux

**Affiliations:** 1 Department of Medicine, Division of Pulmonary, Critical Care, Allergy, and Sleep Medicine,; 2 Department of Surgery, Division of Cardiothoracic Surgery, and; 3 Department of Pathology, University of California San Francisco, San Francisco, California, USA.

**Keywords:** Pulmonology, Cytokines, Fibrosis, Human stem cells

## Abstract

Reciprocal interactions between alveolar fibroblasts and epithelial cells are crucial for lung homeostasis, injury repair, and fibrogenesis, but underlying mechanisms remain unclear. To investigate, we administered the fibroblast-selective TGF-β1 signaling inhibitor epigallocatechin gallate (EGCG) to interstitial lung disease (ILD) patients undergoing diagnostic lung biopsy and conducted single-cell RNA-Seq on spare tissue. Biopsies from untreated patients showed higher fibroblast TGF-β1 signaling compared with nondisease donor or end-stage ILD tissues. In vivo, EGCG downregulated TGF-β1 signaling and several proinflammatory and stress pathways in biopsy samples. Notably, EGCG reduced fibroblast secreted frizzled-related protein 2 (sFRP2), an unrecognized TGF-β1 fibroblast target gene induced near type II alveolar epithelial cells (AEC2s) in situ. Using AEC2-fibroblast coculture organoids and precision-cut lung slices (PCLSs) from nondiseased donors, we found TGF-β1 signaling promotes a spread AEC2 KRT17^+^ basaloid state, whereupon sFRP2 then activates a mature cytokeratin 5^+^ (Krt5^+^) basal cell program. Wnt-receptor Frizzled 5 (Fzd5) expression and downstream calcineurin signaling were required for sFRP2-induced nuclear NFATc3 accumulation and KRT5 expression. These findings highlight stage-specific TGF-β1 signaling in ILD and the therapeutic potential of EGCG in reducing idiopathic pulmonary fibrosis–related (IPF-related) transcriptional changes and identify TGF-β1/noncanonical Wnt pathway crosstalk via sFRP2 as a mechanism for dysfunctional epithelial signaling in IPF/ILD.

## Abstract 2

TGFB1 signaling has a central role in interstitial lung disease and inhibition of TGFB1 by a non-toxic small molecule mitigates damage in a cell model.

## Graphical abstract

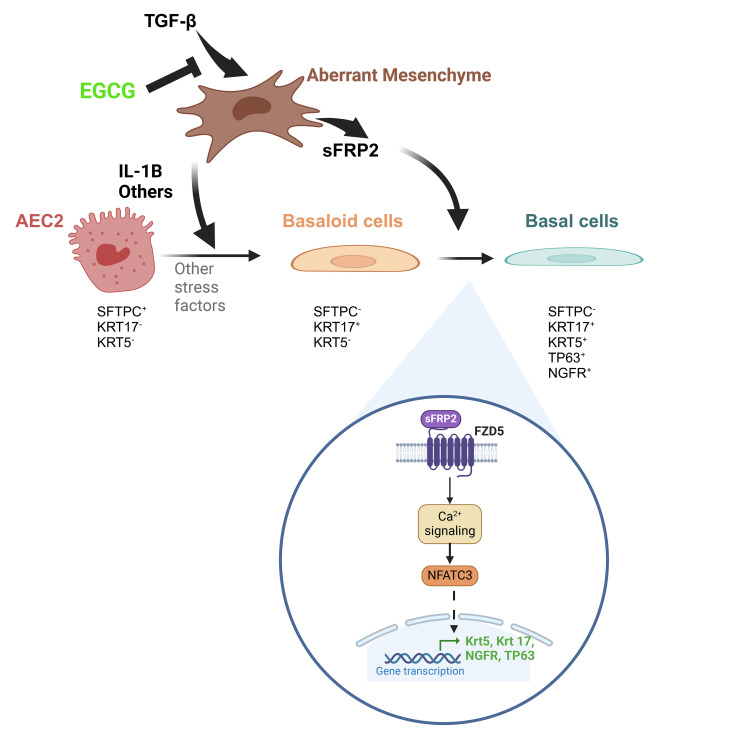

## Introduction

Fibrotic interstitial lung diseases (ILDs), such as idiopathic pulmonary fibrosis (IPF), cause respiratory failure through progressive replacement of lung parenchyma with nonfunctional fibrotic tissue. IPF is widely thought to be caused by epithelial dysfunction, leading to fibroblast activation, extracellular matrix production, and further epithelial dysfunction, causing the usual interstitial pneumonia (UIP) pattern of tissue fibrosis (1–3). Histologically, the UIP pattern is characterized by heterogeneous parenchymal fibrosis, formation of subepithelial fibroblast foci, and alveolar epithelia loss and metaplasia, especially the formation of characteristic honeycomb cysts lined by “bronchiolized” epithelium (4–6), but the molecular and cellular basis of this pathological pattern is unclear. Recent single-cell RNA-Seq (scRNA-Seq) studies of IPF lung tissue confirmed loss of types I and II alveolar epithelial cells (AEC1 and AEC2, respectively) and identified the development of basaloid epithelium (7, 8). AEC2s normally have the dual function of producing pulmonary surfactant and differentiating into AEC1s, which line alveoli (9). However, in IPF, AEC2s can differentiate into cytokeratin 5^+^ (KRT5^+^) basal cells (BCs), normally found lining airways (10). This AEC2-to-BC metaplastic differentiation is regulated by fibroblast signaling and promoted by the pleotropic growth factor TGF-β1, itself a key mediator of tissue remodeling in IPF (10). How these cellular epithelial abnormalities contribute to IPF histopathology and disease progression is not yet fully understood.

We have previously determined that trihydroxyphenolic compounds, such as epigallocatechin gallate (EGCG), cause fibroblast-specific TGF-β1 receptor kinase and lysyl oxidase–like 2 (LOXL2) inhibition, thereby reducing fibroblast activation and collagen crosslinking in fibrotic mouse lungs and in cultured human precision-cut lung slices (PCLSs) derived from IPF patients (11, 12). When taken for 2 weeks, EGCG reduced protein markers of profibrotic signaling in spare diagnostic surgical biopsy tissue from patients with ILD (13). However, there was only limited transcriptional information. Here, we report the results of scRNA-Seq of lung tissue from additional ILD patients who took EGCG for 2 weeks prior to diagnostic surgical biopsy, including comparison with normal donor and end-stage ILD explant lung tissues. The findings provide insights into the scope of TGF-β1–dependent profibrotic mediators increased in IPF and confirm their strong inhibition by EGCG. In the course of this work, secreted frizzled-related protein 2 (sFRP2) was identified as a TGF-β1–upregulated gene in fibroblasts in vivo that directly drives AEC2s toward BC metaplasia ex vivo, implicating sFRP2 as a strong mediator of pathological fibroblast-AEC2 interaction.

## Results

### Fibroblasts from ILD biopsy samples have higher TGF-β1 signaling than IPF explants.

**Figure 1:**
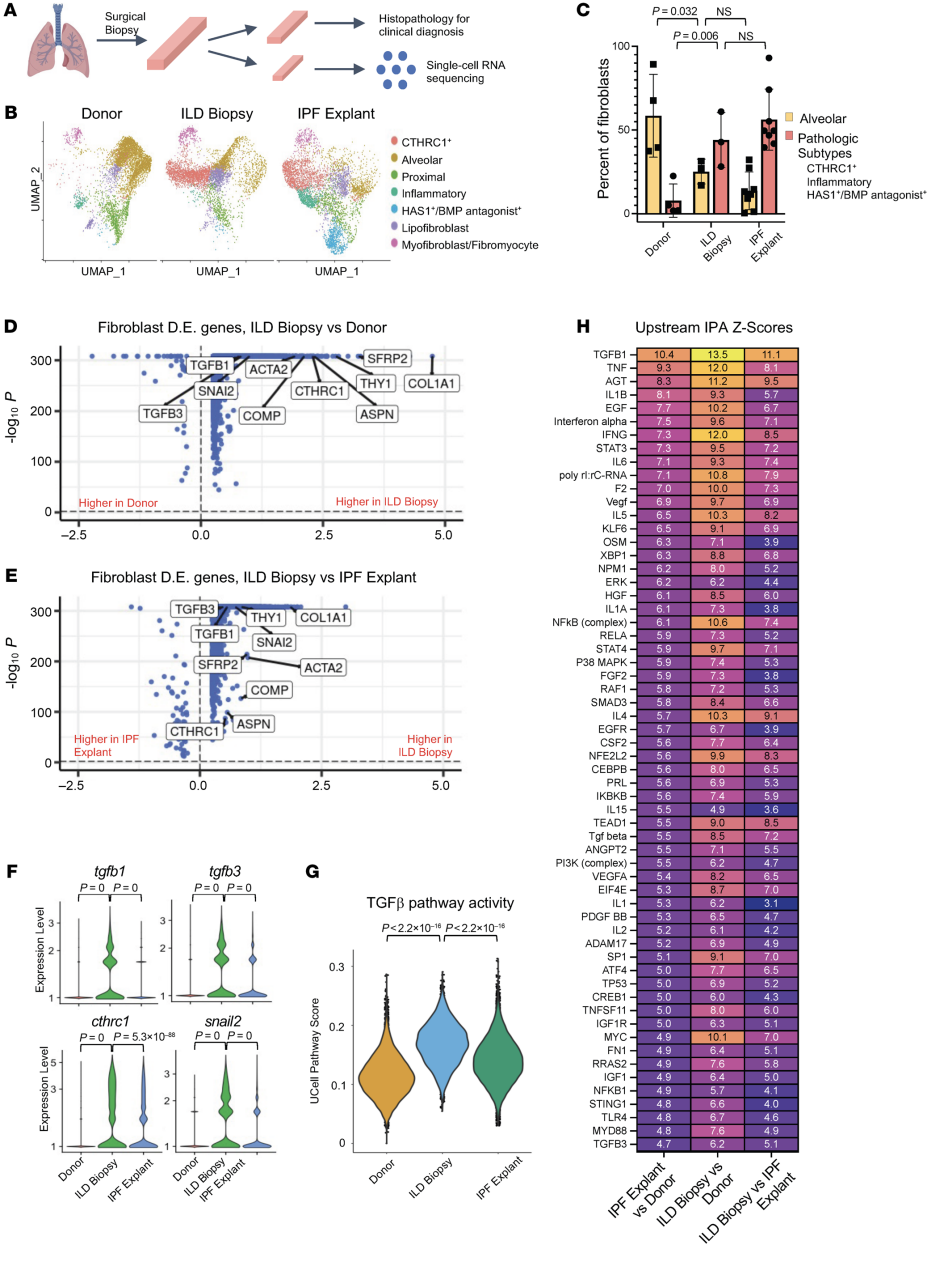
Fibroblasts from ILD biopsies have higher TGF-β signaling than fibroblasts from ILD explants. (**A**) Schematic of processing diagnostic lung biopsies of ILD patients. (**B**) Dimensional reduction plot of fibroblasts from donor (13,856 cells, *n* = 13), ILD biopsy (7,724 cells, *n* = 3), and IPF explant (21,192 cells, *n* = 26) samples. (**C**) Fibroblast subtype composition by sample type. (**D** and **E**) Volcano plots of differentially expressed (D.E.) genes in all fibroblasts from ILD biopsy samples versus donor samples and ILD biopsy samples versus IPF explant samples, with selected TGF-β pathway–related genes labeled. (**F**) Violin plot of selected TGF-β1–related genes in all fibroblasts. (**G**) Single-cell activity of hallmark TGF-β pathway in all fibroblasts. (**H**) Heatmap of *z* scores from pairwise upstream IPA of differentially expressed genes from all fibroblasts. Statistical significance was determined by 2-tailed *t* test (**C**, **F**, and **G**) and MAST with adjustment for multiple comparisons (**D** and **E**).

**Figure 2:**
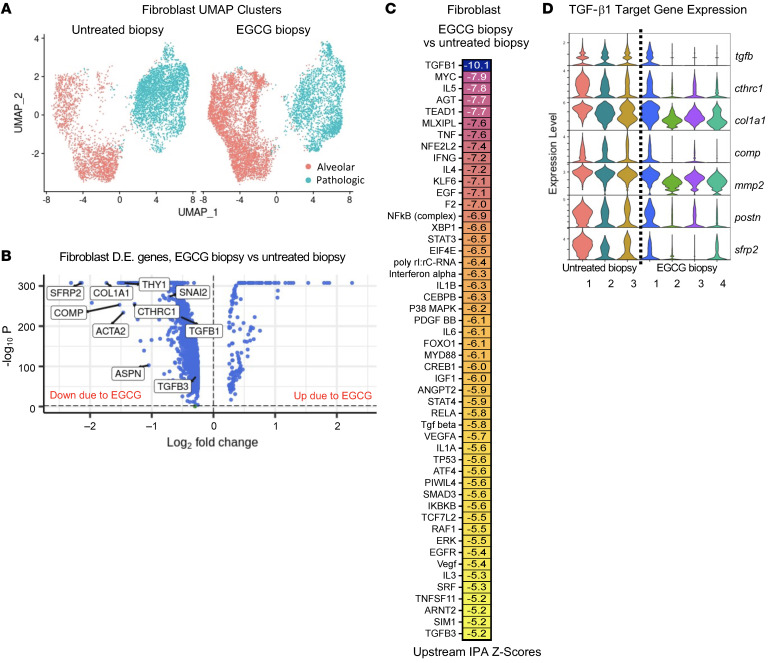
EGCG inhibits TGF-β1 pathway activity in fibroblasts from ILD biopsies and identifies sFRP2 as a downstream target gene. (**A**) Dimensional reduction plot of subsetted and reclustered alveolar (red) and pathologic (blue) (CTHRC1^+^, inflammatory, and HAS1^+^/BMP antagonist^+^) fibroblast subtypes from untreated biopsy (5,225 cells, *n* = 3) and EGCG biopsy (6,773 cells, *n* = 4) samples. (**B**) Volcano plot of differentially expressed genes in alveolar and pathologic fibroblast subtypes from EGCG biopsy versus untreated biopsy samples, with selected TGF-β pathway genes labeled, including sFRP2. (**C**) Heatmap of *z* scores from IPA upstream pathway analysis of differentially expressed genes in alveolar and pathologic fibroblast subtypes from EGCG biopsy versus untreated biopsy samples. (**D**) Violin plots of selected TGF-β pathway genes split by individual biopsy sample.

Eight patients with undiagnosed ILD donated spare diagnostic biopsy tissue, half after taking EGCG (Figure 1A). Patients had mild disease based on most recent pulmonary function testing (Supplemental Table 1; supplemental material available online with this article; https://doi.org/10.1172/JCI174598DS1). We combined our scRNA-Seq files obtained from nondiseased donor lungs (“donor”) samples, untreated biopsy samples (“untreated biopsy”), tissue from ILD patients undergoing lung transplantation for end-stage disease (“IPF explant”), and published samples from the NCBI’s Gene Expression Omnibus database (GEO GSE135893 and GSE132771) (Figure 1B). The integrated transcriptomes of all samples contained various cell types that were identified by classical cell markers (Supplemental Figure 1). Subsequently, we focused our analyses on the transcriptomes of the fibroblasts and epithelial cells (Figure 1 and Figure 2). The sources and cell numbers for the fibroblasts and epithelial cell transcriptomes analyzed in this manuscript are provided in Supplemental Table 2.

We first assessed whether fibroblast transcriptomes evolved with ILD disease progression. Previously established gene markers for annotation of fibroblast subpopulations are detailed in Supplemental Figure 2A. Compared with donor samples, untreated biopsy samples showed reduced alveolar fibroblasts and expansion of previously defined pathological fibroblast subtypes (collagen triple helix repeat containing 1^+^ [CTHRC1^+^], inflammatory, and HAS1^+^/BMP antagonist^+^) (Figure 1C) (8, 10, 14–17). Differential gene expression analysis of untreated ILD biopsy versus control (nondiseased) donor and untreated ILD biopsy versus IPF explant fibroblasts demonstrated transcriptional upregulation of numerous individual genes involved in the TGF-β pathway (Figure 1, D–F, Supplemental Figure 2, B–D, and Supplemental Table 3). Single-cell pathway analysis identified higher TGF-β1 pathway activity in fibroblasts from untreated biopsies than from donor or ILD explants (Figure 1G). Upstream Ingenuity Pathway Analysis (IPA) confirmed upregulation of TGF-β1 signaling in fibroblasts from untreated biopsy samples compared with donor samples, with TGF-β1 itself having the highest *z* score (13.5) of all upstream pathways (Figure 1H and Supplemental Table 3). Notably, several inflammatory and stress pathways also had positive *z* scores above 5: TNFA, IFNG, IL-6, STAT3, AGT, TEAD1, IL1B, P53, p38 MAPK, and MYC. Signaling associated with these pathways was increased in explant fibroblasts relative to donors, but was markedly higher in untreated biopsy than in explants (Figure 1H). Western blot analysis of lung-tissue lysates from independent donor, untreated biopsy, and IPF explant samples demonstrated higher phosphorylated SMAD3 (pSMAD3) expression in untreated ILD biopsies than in IPF explant samples (Supplemental Figure 2, B and C), consistent with the transcriptomic changes. Pan-phosphotyrosine levels were not reduced in control biopsies (Supplemental Figure 2D), indicating that artifact or nonspecific phosphatase activity is unlikely to explain altered pSmad3 levels. These data indicate that fibroblasts from ILD patients with mild disease undergoing diagnostic biopsy have higher TGF-β1 and proinflammatory pathway signaling than either donor tissue or end-stage explant ILD tissue. We next determined to what degree treatment of IPF patients with EGCG for 2 weeks would inhibit TGF-β1 signaling.

### TGF-β signaling in ILD fibroblasts is inhibited by EGCG.

Transcriptomes from biopsy fibroblasts isolated from patients who took EGCG (“EGCG biopsies”) daily for 2 weeks were compared with that of biopsies from patients who were untreated (“untreated biopsies”). Proximal (adventitial) fibroblasts, lipofibroblasts, and smooth muscle cell/myofibroblast transcriptomes were removed from the original data set (Figure 1) to focus on fibroblasts residing near alveoli and/or appearing within the pathology, termed alveolar and pathologic (Figure 2A and Supplemental Figure 3A). Alveolar and pathologic fibroblast subtypes, including CTHRC1^+^, inflammatory, and HAS1^+^BMP antagonist^+^, were subsetted and reclustered. Notably, the alveolar fibroblast cluster in the patient-derived samples comprised cells with canonical alveolar fibroblast markers (e.g., INMT), but also subpopulations with inflammatory gene expression, e.g., CCL2 and CXCL12 (Supplemental Figure 2A), not usually seen in normal alveolar fibroblasts. Dimensional reduction and annotation of subtypes (Figure 2A and Supplemental Figure 3A) showed trends toward altered fibroblast composition due to EGCG treatment, including fewer CTHRC1^+^ and more alveolar fibroblast subtypes (Figure 2A). Comparison of gene expression in fibroblasts in EGCG biopsy versus untreated biopsy samples demonstrated marked downregulation of individual TGF-β1 pathway genes, including types 1 and 6 fibrillar collagens, CTHRC1, and serpine-1 (PAI1) as well as other targets, including sFRP2 (Figure 2, B–D, and Supplemental Table 3). Upstream IPA confirmed downregulation of TGF-β1 signaling due to EGCG treatment as well as downregulation of multiple inflammatory (IL-5, TNFA, IFNG, IL-4, IL-1B, IL-6) and stress signaling pathways (TP53, p38 MAPK, XBP1, MYC) in fibroblasts (Figure 2C and Supplemental Table 3), all of which were upregulated in untreated biopsy fibroblasts compared with donor controls (Figure 1D). Gene expression changes could not be explained by variation among individual samples (Figure 2D). There was substantial overlap in the list of top 100 genes upregulated in untreated biopsy versus donor fibroblasts and the top 100 downregulated genes in EGCG biopsy versus untreated biopsy fibroblasts (Supplemental Figure 3B and Supplemental Table 3), highlighting the central role of TGF-β1 signaling in fibrotic fibroblasts.

### EGCG exposure reduced fibrosis-associated changes in epithelial cells.

**Figure 3:**
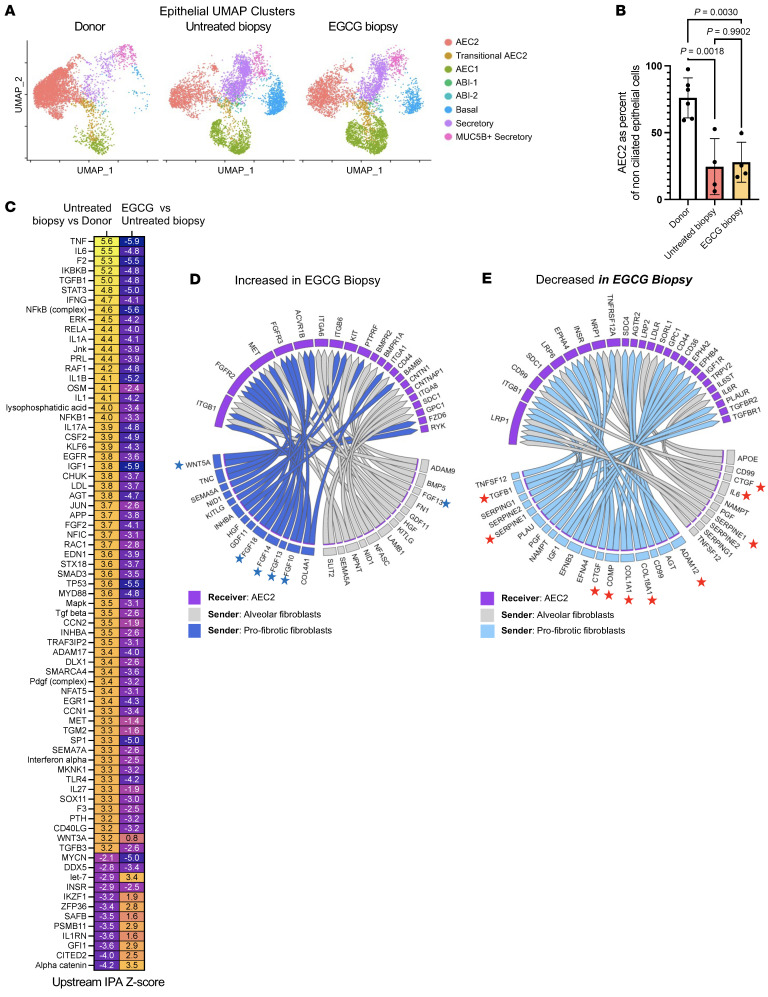
EGCG inhibits IPF-associated changes in biopsy AEC2s. (**A**) Dimensional reduction plot of nonciliated epithelial subtypes from donors (10,127 cells, *n* = 14), untreated biopsy (10,336 cells, *n* = 4), and EGCG biopsy samples (11,585 cells, *n* = 4). (**B**) AEC2s as percentages of nonciliated epithelium. (**C**) Heatmap of selected top up- and downregulated upstream IPA pathways from untreated biopsy versus donor and EGCG biopsy versus untreated biopsy comparisons in AEC2s. (**D** and **E**) NicheNet prediction of differential receptor-ligand signaling from fibroblasts to AEC2s as a result of EGCG. Blue stars highlight AEC2 trophic factors increased in EGCG biopsy samples, and red stars highlight profibrotic pathways decreased in EGCG biopsy samples. Statistical significance was determined by Šídák’s multiple-comparisons test (**B**).

Following dimensional reduction and annotation using known markers of cell type (7, 8, 10, 16), the transcriptomes of epithelial cells were compared across sample type, donor, untreated biopsy, and EGCG biopsy (Figure 3A and Supplemental Figure 3C). A marked loss of AEC2s was already observed in untreated biopsy samples, as previously reported in IPF explant lungs (7, 8) (Figure 3B; note that AEC2s are shown as percentage of nonciliated epithelium). Moreover, an upstream IPA on differentially expressed genes in AEC2s from untreated biopsies compared with nondiseased donors had positive *z* scores for numerous inflammatory and stress pathways, e.g., IL-6, TNF-α, NFKB, JNK, TP53, DDX5, and p38 MAPK, as well as for TGF-β1. Even though the 2-week EGCG exposure did not reverse the loss of AEC2 seen in untreated biopsy, a near-complete pattern reversal was measured on the mentioned differentially expressed genes in AEC2s (Figure 3C and Supplemental Table 3). The NicheNet receptor-ligand signaling prediction algorithm was used to infer altered signaling from fibroblast ligands to AEC2 receptors due to EGCG. Notably, signaling from numerous AEC2-supporting niche factors, such as multiple FGFs, WNT5A, BMP5, and HGF, was increased in the EGCG-treated group (Figure 3D). Conversely, fibrosis-associated signaling pathways, such as collagens, CTGF, IL-6, SERPINE1/PAI1, COMP, APOE, and TGF-β1, were all predicted to be decreased in EGCG-treated samples (Figure 3E). EGCG requires LOXL2 for its activation as a TGF-β1 kinase inhibitor. LOXL2 is not expressed in epithelial cells, including AEC2s, and hence EGCG has no discernible direct effect on AEC2s (11). These findings suggest that inhibition of fibroblast TGF-β1 signaling by EGCG reduces fibroblast-mediated changes in alveolar epithelial cells.

### sFRP2 is a fibroblast-specific TGF-β1 gene target in IPF.

**Figure 4:**
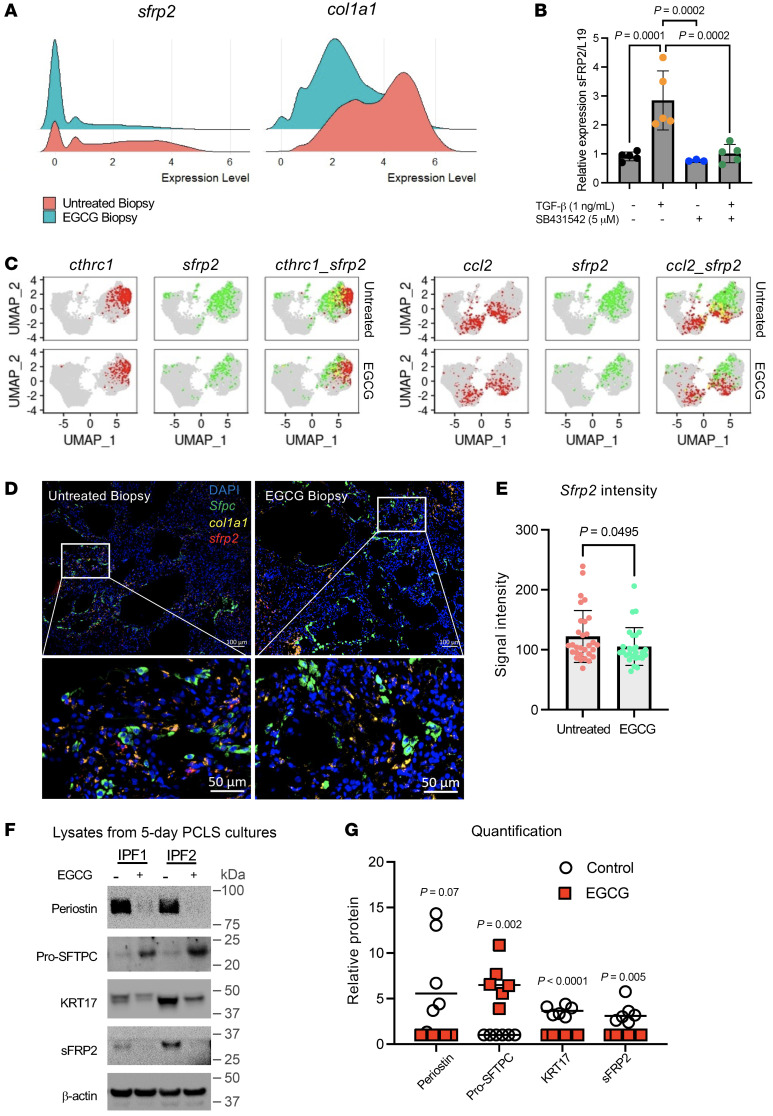
Impact of EGCG on distribution of sFRP2 expression in fibroblast subpopulations, biopsy tissues, and PCLS cultures. (**A**) Ridge plot of *sfrp2* and *col1a1* gene expression in alveolar and pathologic fibroblast subtypes from untreated and EGCG biopsy samples. (**B**) Relative expression of *sfrp2* mRNA in human fibroblasts treated with TGF-β1 (1 ng/ml) and/or TGF-β inhibitor SB4331542 (5 μM) for 48 hours (*n* = 5). (**C**) Feature plots of *sfrp2* gene expression (green) in various fibroblast subsets characterized by *cthrc1* or *ccl2* gene expression (red). Cells expressing both *sfrp2* and *cthrc1* or *ccl2* are indicated in yellow. Additional fibroblast markers are shown in Supplemental Figure 4B. (**D** and **E**) RNA in situ hybridization was performed for *col1a1* (yellow) and *sfrp2* (red) genes in untreated and EGCG biopsies (**D**). The signal intensity of *sfrp2* was quantified for each image (**E**). Representative images of *n* = 5 samples per group, 4–6 images per sample. Original magnification, ×100 (top images). Bottom images represent a region of interest as indicated by white rectangle. (**F** and **G**) PCLSs from IPF lung donors cultured for 7 days with EGCG (1 μM) and analyzed by Western blot. Additional samples are shown in Supplemental Figure 5C. (**G**) Graphical representation of the level of expression of selected proteins for all samples (see Supplemental Figure 5C). *n* = 6. Statistical significance was determined by the Kruskal-Wallis test (**B**) and 2-tailed *t* test (**E** and **G**).

Analysis of the fibroblast genes downregulated by inhibition of TGF-β1 signaling identified sFRP2 as one of the most downregulated genes, with a log_2_ fold change of –2.1 (Figure 2D, Figure 4A, and Supplemental Table 3). We confirmed that sFRP2 is a TGF-β1 target gene, based on strong and specific upregulation of *sFRP2* mRNA by TGF-β1 in primary human lung fibroblasts that was suppressed when a TGF-β1 inhibitor was added to the culture (Figure 4B).

Moreover, among the 5 known human sFRPs, only sFRP2 is strongly upregulated by TGF-β1 (Supplemental Figure 4A), and inspection of several online scRNA-Seq files revealed that sFRP2 expression is specific to fibroblasts in ILD (7, 8, 17–19) and Rosas and Kropski online scRNA-Seq files at http://www.ipfcellatlas.com We next determined which subpopulations of fibroblasts expressed sFRP2 in fibrotic lungs.

*sfrp2* Expression was found in several subpopulations of fibroblasts within the alveolar compartment, as indicated by superimposition of *sfrp2* expression on various feature plots within the alveolar and pathological fibroblast clusters (Figure 2) from untreated or EGCG-treated patients (Figure 4C and Supplemental Figure 4B). Virtually all of the *sfrp2* expression colocalized with *col1a1* (Supplemental Figure 4B) and partially with *cthrc1* and the inflammatory marker *ccl2*, all of which were suppressed by EGCG treatment. Although there was little colocalization of *sfrp2* and the alveolar fibroblast markers *inmt* and *tcf21*, the expression of these markers was also obviously suppressed in fibroblasts of EGCG-treated patients (Supplemental Figure 4B). The overall intensity of *sfrp2* signal by in situ hybridization within the alveolar space was substantially decreased in EGCG-treated patients (Figure 4, D and E), consistent with the scRNA expression data (Figure 2D). To further define the spatial distribution of *sfrp2* expression, we measured the average distance from *sftpc^+^* cells to *sfrp2^+^* cells using in situ hybridization and found clusters of *sfrp2^+^* cells to be substantially correlated with proximity to *sftpc^+^* AEC2s and *krt17^+^sftpc^+^* alveolar-basal intermediate (ABIs) cells (Supplemental Figure 5, A and B). Finally, we confirmed the reduced expression of sFRP2 by Western blot protein analysis of PCLSs from 6 IPF explants cultured without or with EGCG for 7 days. EGCG effected marked decreases in fibroblast (periostin, sFRP2) and epithelial (KRT17) genes that are upregulated in IPF as well as increased surfactant protein C (SFTPC) expression (Figure 4, F and G, and Supplemental Figure 5C).

sFRP2 is an interesting protein relevant to pulmonary fibrosis in several respects. Prior studies have found that sFRP2 is a fibroblast-specific protein and its expression increases with age (16, 18). sFRP2 has been linked to fibrosis in several experimental systems (19–21). Interestingly, sFRP2 is found in gene signatures of IPF patients analyzed by GWAS or other linkage analyses (22, 23), suggesting a role in IPF pathobiology. Collectively, these findings and the findings detailed above prompted us to explore the functional effects of fibroblast sFRP2 expression ex vivo.

### SFRP2 induces expression of basal genes in cultured human AEC2 cells.

**Figure 5:**
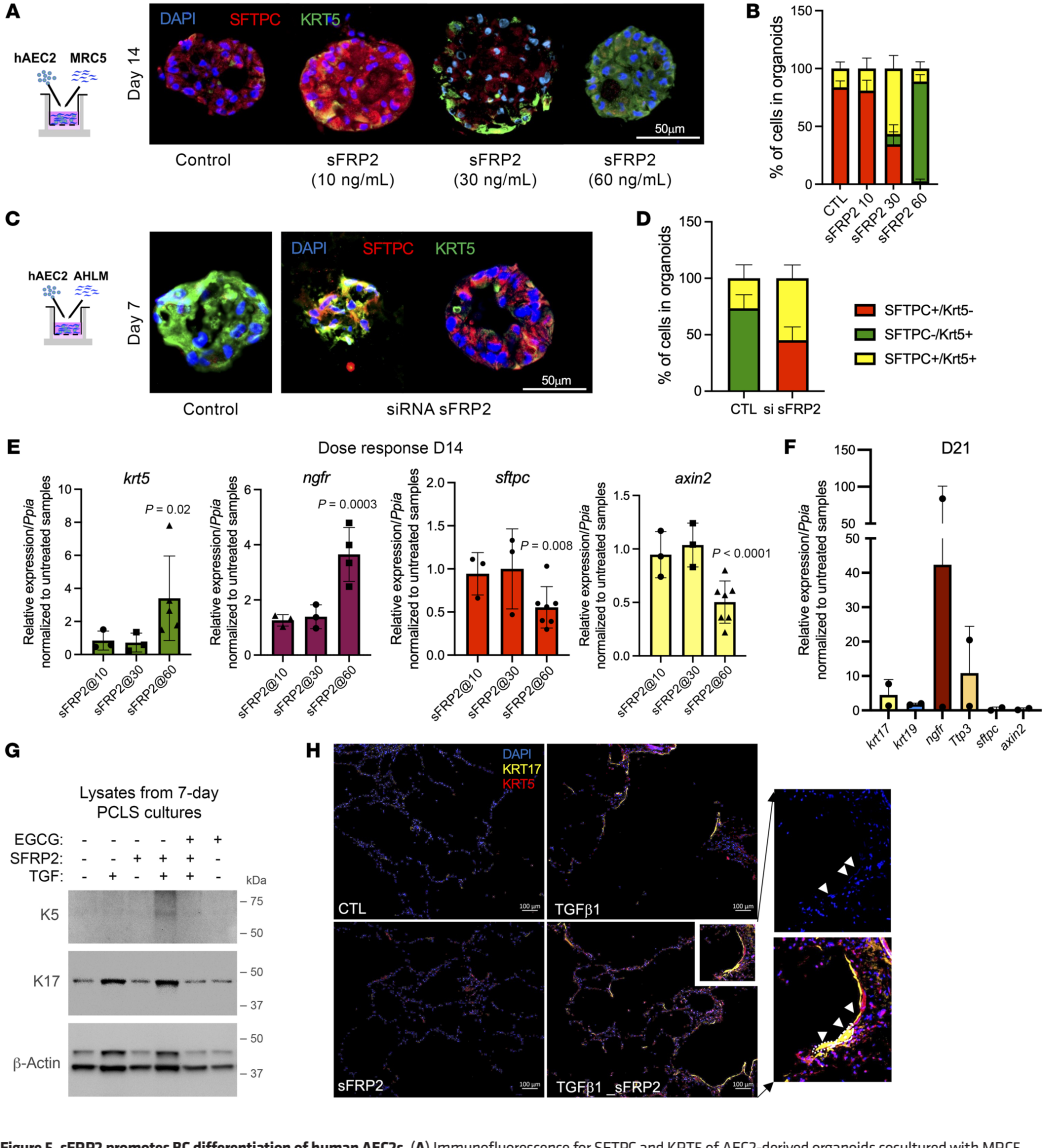
sFRP2 promotes BC differentiation of human AEC2s. (**A**) Immunofluorescence for SFTPC and KRT5 of AEC2-derived organoids cocultured with MRC5 cells treated with sFRP2 for 14 days. Representative of *n* = 5 biological replicates. The experiment was performed in 3 technical triplicates, and data from 3 technical replicates are counted as 1 biological replicate. Original magnification, ×200. (**B**) Percentages of SFTPC^+^KRT5^–^, SFTPC^+^KRT5^+^, and SFTPC^–^KRT5^+^ cells in day-14 AEC2s plus MRC5 organoids treated with sFRP2. (**C**) Immunofluorescence of AEC2-derived organoids cocultured with AHLM after sFRP2 silencing. Original magnification, ×200. (**D**) Percentages of SFTPC^+^KRT5^–^, SFTPC^+^KRT5^+^, and SFTPC^–^KRT5^+^ cells in day-7 AEC2s plus AHLM^sfrp2neg^ organoids. Data are presented as means of *n* = 4 biological replicates. (**E**) Levels of expression of *krt5*, *ngfr*, *sftpc*, and *axin2* mRNA in EPCAM^+^ cells isolated from day-14 AEC2s plus MRC5 organoids treated with sFRP2. *n* = 3 biological replicates for sFFRP2 (10 and 30 ng/ml) and *n* = 4–7 biological replicates for sFRP2 (60 ng/ml). (**F**) Expression of genes characteristic for BCs in EPCAM^+^ cells isolated from day-21 AEC2s plus MRC5 organoids treated with sFRP2 (60 ng/ml). *n* = 2 biological replicates. (**G** and **H**) PCLSs from nondiseased donors were cultured and treated with or without TGF-β1 (2 ng/ml), with or without sFRP2 (60 ng/ml), and with or without EGCG (1 μM) for 7 days. (**G**) Lysates were blotted for KRT5 and KRT17. *n* = 3 biological replicates. (**H**) Immunofluorescence of KRT5 and KRT17. Representative of *n* = 3 independent experiments. Original magnification, ×100. Region of interest is presented as an insert (white rectangle) to show elongation of nuclei (DAPI) and cell morphology, outlined as a dotted line in insert as indicated by white arrowheads. Statistical significance was determined by mixed-effects analysis followed by Tukey’s multiple-comparisons test (**B** and **D**), Dunnett’s multiple comparisons test (**E**), and 2-tailed *t* test (**F**). *P* values are reported in Supplemental Table 4 for **B** and **D**.

We first tested the impact of recombinant sFRP2 on the fate of AEC2s in organoid cocultures with the embryonic fibroblast MRC5 as feeder cells, as prior work has established these cocultures maintain the integrity and support expansion of AEC2 colonies (8). Low concentrations of sFRP2 (10 ng/ml) supported AEC2 integrity with SFTPC expression, whereas progressively higher concentrations effected transdifferentiation of the AEC2 cells to KRT5^+^ basal-like cells (Figure 5A). By day 14 of the coculture, all the organoids derived from AEC2-MRC5 treated with 60 ng/ml of sFPRP2 contained KRT5^+^ cells, with some cells still expressing SFTPC, whereas all the organoids in the AEC2-MRC5 coculture treated with 10 ng/ml of sFRP2 contained SFTPC^+^ cells, with few expressing KRT5 (Figure 5B and Supplemental Table 4). Treatment with 30 ng/ml induced an intermediate phenotype, with a similar distribution of cells still expressing SFTPC and cells expressing KRT5 (Figure 5, A and B). Given the consistent promoting effects of exogenous sFRP2 on AEC2 transdifferentiation, we asked whether fibroblast expression of sFRP2 was required for fibroblast-dependent expression of KRT5 in AEC2s in cultured organoids. The expression of sFRP2 in primary adult human lung mesenchyme (AHLM) was silenced immediately prior to formation of organoids, as AHLM is known to drive basal metaplasia of AEC2s in coculture organoids (10). We confirmed silencing of *sfrp2* in AHLM transfected with *sfrp2* siRNA versus control siRNA (0.22 ± 0.1-fold expression versus control). The *sfrp2* silencing markedly attenuated both loss of SFTPC and gain of KRT5 protein levels in AEC2s in coculture organoids, as compared with organoids formed with AHLM treated with control siRNA (Figure 5C and Supplemental Table 4). However, some colonies developed mixed phenotypes, comprising both SFTPC^+^ and KRT5^+^ cells, whereas others were virtually devoid of a KRT5 transition (Figure 5, C and D). These findings confirm a critical role of mesenchymal sFRP2 in regulating AEC2 cell fate.

To confirm that sFRP2 treatment promoted basaloid differentiation, we isolated RNA from 14-day organoids similar to that shown and quantified mRNA for *krt5*, *ngfr*, *axin2*, and *sftpc*. As shown in Figure 5E, sFRP2 promoted *krt5* and *ngfr* mRNA expression and decreased *axin2* and *sftpc* mRNA levels. By 21 days, sFRP2 induced high levels of *ngfr*, *tp63*, and *krt17* (Figure 5F). Collectively, sFRP2 reduced canonical Wnt signaling and promoted transdifferentiation of AEC2 to basal-like cells.

As a second approach, PCLSs were prepared from nondiseased donor lungs and cultured without or with sFRP2 for 7 days to investigate its effect on basal gene expression in AEC2s. No KRT5 or KRT17 expression was observed on freshly fixed nondiseased lung section (data not shown). The addition of TGF-β1 (2 ng/mL) promoted the expression of KRT17 in AEC2s as well as an elongated epithelial morphology by day 3 (not shown) that persisted through day 7, but little or no KRT5 (Figure 5, G and H, and Supplemental Figure 6). Combined treatment with TGF-β1 and sFRP2 (60 ng/mL) induced a strong expression of KRT5 detected by both Western blot analysis of lung slices and immunostaining in very elongated Krt17^+^ cells by immunofluorescent staining (Figure 5, G and H, and Supplemental Figure 6). The insert reveals colocalization of KRT17 and KRT5 in elongated AEC2-derived alveolar cells consistent with BC cytoplasmic extensions (24). Furthermore, treatment with EGCG reduced the expression of KRT17 and KRT5, suggesting that TGF-β1 promotes KRT17 expression indirectly through the mesenchyme because EGCG does not inhibit TGF-β1 signaling in epithelial cells (11).

### sFRP2 acts directly on AEC2s through the Frizzled 5 receptor to activate noncanonical Wnt signaling and promote basal gene expression.

**Figure 6:**
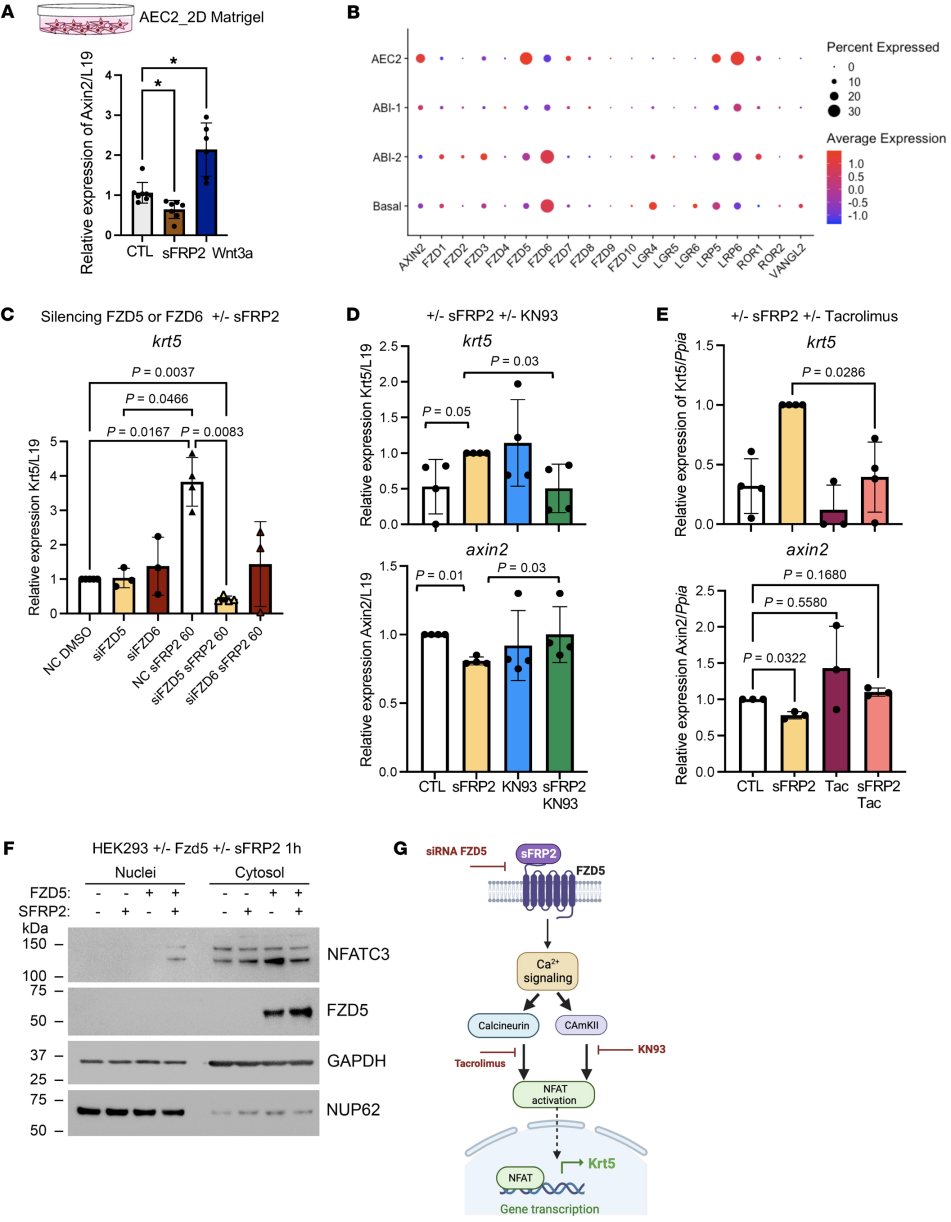
sFRP2 acts on AEC2s through the Fzd5 receptor to promote noncanonical Wnt signaling. (**A**) *axin2* gene expression in AEC2s cultured for 48 hours with sFRP2 (60 ng/ml) or Wnt3a (100 ng/ml). *n* = 4–6 biological replicates. *P < 0.05 (**B**) Dot plot of Frizzled receptors and related coreceptors from selected epithelial cells from donor, control biopsy, and ILD explant samples. (**C**) *krt5* mRNA levels expressed in AEC2s were measured after silencing the expression of *fzd5* or *fzd6*. The silenced *fzd5* and silenced *fzd6* AEC2 cells were subsequently treated with 60 ng/ml of sFRP2 for 48 hours. *n* = 3 independent biological replicates. (**D**) Levels of expression of *krt5* and *axin2* mRNA were measured in AEC2 cells treated with CaMKII inhibitor KN93 (1 μg/ml^–1^) with or without sFRP2 (60 ng/ml) for 48 hours. (**E**) Levels of expression of *krt5* and *axin2* mRNA were measured in AEC2 cells treated with tacrolimus (1 μM) with or without sFRP2 (60 ng/ml) for 48 hours. (**F**) Western blot indicating the presence of NFATC3 in nuclei extract from HEK293 cells transfected with FZD5 plasmid followed by treatment with sFRP2 (30 ng/ml) for 1 hour. *n* = 3 biological replicates. (**G**) Schematic of the sFRP2-FZD5 signaling pathway promoting the expression of KRT5 in AECs. Created with BioRender. Statistical significance was determined by Kruskal-Wallis (**A**), Tukey’s test (**C**), or Dunnett’s test (*axin2* in **E**) and Mann–Whitney *t* test (**D**, *krt5* in **E**).

To determine whether sFRP2 directly acts on AEC2, we cultured freshly isolated human AEC2 cells on top of Matrigel without fibroblast support. Because sFRP2 suppressed the canonical Wnt target *axin2* in organoids (Figure 5C), we compared the effects of sFRP2 and Wnt3a on *axin2* levels of cultured AEC2s (Figure 6A). As expected, sFRP2 suppressed whereas Wnt3a induced *axin2* mRNA expression in isolated AEC2s, confirming that sFRP2 does not promote detectable canonical Wnt signaling in these cells. Immunofluorescent staining of parallel AEC2 cell cultures showed that exposure to sFRP2 (60 ng/ml) caused loss of SFTPC and induction of KRT5^+^ in AEC2s (Supplemental Figure 7A). Furthermore, quantitative real-time PCR (qPCR) analysis indicated that sFRP2 exposure both induced *krt5* and suppressed *axin2* mRNA (Supplemental Figure 7B). These findings indicate that sFRP2, which is induced in fibroblasts by TGF-β1, acts directly on AEC2 cells to promote their transdifferentiation toward basal-like cells in organoid cocultures.

To explore potential receptors for sFRP2 on AEC2s, we examined Frizzled receptor and coreceptor expression in our epithelial scRNA data (Figure 6B). Frizzled 5 (Fzd5) is the Frizzled with the highest expression in AEC2s, but Fzd6 is the most expressed Frizzled receptor in BCs. As sFRP2 was recently reported to bind to human endothelial FZD5 receptors and promote noncanonical signaling through a calcineurin/NFAT signaling pathway (25), a similar process may occur in human AEC2s. We therefore tested the role of FZD5 in sFRP2-induced basal gene expression in AEC2s by knocking down *fzd5* via siRNA and culturing on top of Matrigel with sFRP2 (60 ng/ml). The *fzd5* knockdown in AEC2s almost completely blocked the induction of *krt5* mRNA expression by sFRP2, while *fzd6* knockdown did not have a significant effect (Figure 6C). Although not statistically significant, the trend toward reduced *krt5* mRNA after *fzd6* knockdown may reflect the switch in relative levels of FZD5 and FZD6 during differentiation of AEC2s toward BCs (Figure 6B). Knockdown was confirmed in transfected AEC2s, as siRNA against *fzd5* reduced its expression to 0.37 ± 0.14-fold relative to control and siRNA against *fzd6* reduced its expression to 0.46 ± 0.07-fold relative to control.

To further test the role of noncanonical Wnt signaling as the mechanism of sFPR2-induced basal gene expression, we examined the effects of 2 mechanistically distinct inhibitors of the Ca^2+^ activation arm of noncanonical Wnt signaling: KN93, a CaM kinase II inhibitor, and tacrolimus (26). KN93 blocked upregulation of *krt5* mRNA and downregulation of *axin2* mRNA in 2D cultures of AEC2s stimulated with sFRP2 (60 ng/ml) (Figure 6D). Similarly, tacrolimus, a direct inhibitor of calcineurin activity, completely blocked sFRP2-induced *krt5* expression and restored *axin2* levels to those of untreated controls (Figure 6E). Because dephosphorylation of NFAT by calcineurin is the key step that promotes its nuclear accumulation and signaling, we attempted to demonstrate nuclear NFAT in sFRP2-stimulated primary AEC2 cells. However, we found the basal levels of NFATC3 (the major form of NFAT in human AEC2s) to be low, and we were unable to detect nuclear NFAT biochemically. We therefore turned to HEK293 cells, which have higher basal NFATC3 and, importantly, no expression of FZD5. We observed that sFRP2 in 3 independent experiments induced nuclear NFATC3 accumulation and this was completely dependent on prior transfection of the cells with FZD5 cDNA, confirmed by Western blotting (Figure 6F). A schematic summarizing the effect of sFRP2 on AEC2 cell fate through FZD5 signaling and calcineurin-dependent NFAT nuclear accumulation is shown in Figure 6G.

## Discussion

Extensive evidence implicates TGF-β1 signaling as a causative driver of fibrotic processes, including IPF and other fibrotic ILDs. The findings reported here confirm at a transcriptional level our prior finding demonstrating that selective inhibition of fibroblast TGF-β1 signaling by EGCG attenuates profibrotic signaling at a protein level. We find robust evidence for decreased TGF-β1 signaling due to EGCG, based on changes in expression of individual genes known to be linked to TGF-β1 signaling and by gene pathway analyses. The findings also provide insights relevant to the mechanisms underlying fibrotic ILDs. Variable TGF-β1 signaling activity in ILD patient tissues at different disease stages has not previously been reported and suggests that TGF-β1 may have a different effect in patients with mild disease as compared with late- or end-stage disease. This is consistent with evidence that matrix accumulation per se can become a TGF-β1–independent driver of more matrix accumulation, in part perhaps related to activation of YAP/TAZ signaling (27, 28). We cannot distinguish between the possibility that TGF-β1 activation and downstream signaling diminishes with late-stage disease and the possibility that this is mainly relative to matrix-dependent signals and infiltration of inflammatory cells that become more robust as disease progresses. In either case, our data suggest that fibroblast TGF-β1 activity may have an especially important role in the development of early tissue-level changes. Further study will be needed to better understand how TGF-β1 activity and other signaling pathways contribute to distinct phases of IPF pathology. This may well affect the choice and testing of drugs to attenuate disease progression.

A surprising finding in our studies is the suppression of epithelial proinflammatory and stress signaling by selective inhibition of fibroblast TGF-β1 signaling. While IPA identified upregulation of many inflammatory pathways in fibroblasts and epithelial cells from untreated biopsy patients and their inhibition by EGCG, it is important to note that these pathways share some common downstream effector mechanisms, which suggests a network of overlapping feed-forward interactions rather than multiple pathways acting or inhibited in isolation. Nonetheless, the overall outcome of EGCG exposure appears to attenuate the activity of multiple proinflammatory and stress effectors and thereby to promote the maintenance of AEC2 differentiation. This conclusion is independently supported by our finding that EGCG reduced KRIT17 (a basaloid cell marker) and increased SFTPC protein in lysates of 5-day PCLS cultures derived from IPF patient explants (Figure 4). What are the underlying mechanisms? One possibility is that EGCG has unrecognized effects on inflammatory signaling pathways identified here, either as a result of or in addition to its known inhibition of TGF-β1. However, we favor the view that EGCG effects on the epithelium can mainly be explained by its suppressing secreted fibroblast profibrotic mediators that target the epithelium, e.g., TGF-β1 itself, extracellular matrix proteins, and others implicated in the NicheNet analysis (Figure 3, D and E) and by our findings here with sFRP2.

sFRP2 structurally resembles the ectodomain of a Frizzled receptor and was originally identified as a Wnt antagonist (29, 30), presumably by acting as a decoy for Wnt ligand binding. Subsequently, positive effects of sFRP2 on canonical Wnt signaling have been identified (31), and sFRP2 has been found to interact with FZD5 and FZD7 to promote noncanonical Wnt activity (25, 32). Canonical Wnt activity has been repeatedly linked to maintenance of AEC2 fate (33, 34), but is also known to be increased in IPF (35, 36) despite AEC2 loss. sFRP2 expression has previously been linked to TGF-β1 signaling in colonic cancer fibroblasts where inhibition of PKCδ expression by TGF-β1 induced cell-autonomous Sox2 expression and subsequent direct upregulation of sFRP1 and sFRP2 (37). However, in primary normal human lung fibroblasts, TGF-β1 selectively induced sFRP2 and not sFRP1 (Supplemental Figure 4A), suggesting different regulation. It remains to be defined whether SOX2 is also involved in this process. In any case, in the human lung fibroblast system studied here, sFRP2 does not induce canonical Wnt signaling when compared with Wnt3a (Figure 6A), but promotes the noncanonical FZD5 Ca^2+^ signaling pathway (Figure 6).

Our studies of AEC2-fibroblast coculture organoids and PCLSs (Figure 5, F and G) exposed to TGF-β1 without or with sFRP2 shed light on the process of AEC2 cell transdifferentiation to BCs, a prominent feature of human fibrotic lung diseases (38).

We found that TGF-β1 signaling alone promotes a spread, AEC2-derived SFTPC^–^KRT17^+^ basaloid state, perhaps to cover denuded alveolar walls, as recently suggested by studies of AT1 deletion in mice (39). This finding also indicates uncoupling of Krt17 and Krt5 expression, the staged maturation and requirement for additional signaling suggesting a distinct and likely transient function of basaloid cells in alveolar repair. Whether AEC2-derived BCs and persistent basaloid cells become pathological or are themselves capable of resuming an AEC2 or AEC1 state requires further study (7, 40).

There are limitations to this study. The number of patient samples processed and analyzed is small, though the total cells profiled in each group provided substantial statistical power and we were unable to find evidence that variation among individual samples was responsible for our findings. Another limitation is that while we are able to link sFRP2 signaling through FZD5 to calcineurin and nuclear NFAT in a pathway to BC differentiation, we have no evidence for a direct connection between nuclear NFAT and activation of the BC transcriptional state. Thus, we are uncertain how NFAT signaling evolves to activate mature BC reprogramming. Further elucidation of this pathway will require more study. Finally, an additional limitation is the lack of capture of immune cells from the biopsy samples for scRNA-Seq, thereby precluding any assessment of TGF-β1–mediated changes in fibroblast inflammatory markers on the immune system.

In summary, in this manuscript, we confirm the central role of TGF-β1 signaling in ILD, finding elevated levels in patients with mild clinical disease. We confirm inhibition of TGF-β1 signaling by EGCG and found multiple downstream likely beneficial effects, such as reduced profibrotic signaling in fibroblasts and reduction in IPF-associated changes in AEC2s. We identify a fibroblast-dependent pathway required to promote epithelial metaplasia prominent in IPF pathobiology. These results support a potential therapeutic role for EGCG in IPF, which is relevant as there is now an active phase 1/2 trial in IPF patients (ClinicalTrials.gov NCT05195918). Improved treatment in IPF is a pressing clinical need.

## Methods

### Sex as a biological variable

Sex was not considered as a biological variable. Patients of both sexes were enrolled in the study. Since consenting study patients were selected solely based on clinically indicated lung biopsies, the total number of subjects was too small to allow for grouping by sex.

### Human lung tissue

Tissue from normal lungs declined for transplantation and from IPF patients undergoing transplantation were deidentified and donated to research. Patients from the UCSF Interstitial Lung Disease Clinic who were referred for diagnostic surgical lung biopsy were identified and enrolled in a convenience cohort; they took 600 mg of EGCG by mouth once daily for 2 weeks prior to biopsy, and a spare biopsy was obtained at the time of surgical resection. Note that for control biopsy 3, FACS purification of fibroblasts yielded few cells for unclear reasons, so scRNA-Seq on this sample was only performed on epithelial cells.

### Lung tissue processing and FACS

#### Lung digestion, FACS.

A single cell preparation of normal, ILD-explant, or biopsy tissues was made using mechanical disruption and enzymatic digestion (dispase, 15 IU.ml^–1^, and collagenase, 225 U.ml^–1^), as previously described (10). For AEC2 isolation, FACS was performed on digested donor lung tissue for live EPCAM^+^CD11b^–^CD31^–^CD45^–^HT_2_-280^+^ cells (Supplemental Table 7). For samples used in scRNA-Seq, FACS was performed on digested lung tissue for live epithelial (EPCAM^+^CD11b^–^CD45^–^CD31^–^) and mesenchymal (EPCAM^–^CD11b^–^CD45^–^CD31^–^) cells, which were combined 1:1 for processing with the 10x Genomics Chromium platform and sequenced on an Illumina NovaSeq 6000 machine.

### scRNA analysis

Raw sequencing results were processed with the 10x Genomics Cell Ranger pipeline and analyzed with Seurat, version 4, including normalization with SCTransform, version 2, and integration with the FastMNN packages (41–43). All cells from each sample were initially integrated, followed by iterative subsetting. Clustering was performed by increasing the resolution until differences were not biologically meaningful, and then cell annotation was performed using previously described markers (7, 8, 10, 14–17). Corrected gene counts were used for feature and violin plots. Seurat object cell composition was analyzed in samples containing 500 or more cells. Differential gene expression was performed on SCTv2-normalized counts using the Model-based Analysis of Single-cell Transcriptomics (MAST) method (44). IPA (45) was done on differentially expressed genes using log_2_ fold change thresholds of ±0.25 and adjusted *P* values of less than 0.05. Single-cell pathway activity analysis was done using the UCell package (46) with MSigDb gene sets (47). Comparative receptor-ligand signaling analysis was performed with NicheNet (48) using normalized RNA counts.

### In vitro and ex vitro models

#### Cell culture.

MRC5 fibroblasts (catalog CCL-171, ATCC) and adult human mesenchymal cells were cultured in DMEM (catalog 11965092, Thermo Fisher) with 10% fetal bovine serum (catalog SH883IH2540, Fisher Scientific), 1% glutamax (catalog 35050-61, Gibco, Thermo Fisher Scientific), 1% HEPES (catalog 5630-080, Gibco, Thermo Fisher Scientific), and 1% penicillin/streptomycin (catalog 10378016, Gibco, Thermo Fisher Scientific). Cells were used within the first 5 passages of either being received from ATCC for MRC5 cells or being isolated from donor lungs for mesenchymal cells. Where applicable, TGF-β1 (catalog 100-21, Peprotech, 1 and 4 ng/ml), SB431442 (catalog 1614/1, Tocris, 5 mM), a TGF-β1 inhibitor (49), sFRP2 (catalog 6838-FR, R&D Systems, 60 ng/ml), tacrolimus (catalog AAJ6357AMF, Thermo Fisher, 1 μM), Wnt3a (catalog 5036-WN-010, R&D, 100 ng/ml), and KN93 (catalog 422711-1MG, MilliporeSigma, 1 μM) were added to the medium after 24 hours for 48 hours.

#### siRNA.

siRNA probes targeting *sfrp2* in human mesenchymal cells and *Fzd5* and *Fzd6* in AEC2 cells were obtained from Thermo Fisher (Supplemental Table 6). In brief, 20,000 hAEC2s were incubated for 4 hours in Opti-DMEM (catalog 31985062, Fisher Scientific) with 1 pmole of siRNA probes using Lipofectamine RNAiMAX (catalog 13778, Invitrogen). Cells were then plated on growth factor–reduced Matrigel (catalog CB-40230A, Thermo Fisher) cultured in small airway basal medium (SABM) (catalog CC-3118, Lonza) with BPE low protein, insulin, transferrin, retinoic acid, epinephrin, triiodothyronine, and epidermal growth factor as per the SAGM BulletKit (catalog CC-3118, Lonza) supplemented with KGF (catalog 251KG01050, R&D, 100 ng/ml), 5% charcoal-treated FBS (catalog 12676011, Thermo Fisher), and 1% penicillin/streptomycin for 48 hours.

#### Organoid assay.

AEC2s and MRC5 fibroblasts or mesenchymal cells with or without silenced *sfrp2* were cocultured (5,000 AEC2s: 30,000 fibroblasts/mesenchymal cells per well) in modified MTEC medium diluted 1:1 in growth factor–reduced Matrigel (catalog CB-40230A, Thermo Fisher). Modified MTEC culture medium is composed of SABM with insulin, transferrin, bovine pituitary extract, retinoic acid, and EGF as per the SAGM BulletKit and 0.1 μg/ml^−1^ cholera toxin (catalog C8052, MilliporeSigma), 5% charcoal-treated FBS, and 1% penicillin/streptomycin. The cell suspension–Matrigel mixture was placed in a Transwell and incubated with 10 μM ROCK inhibitor (catalog 72252, STEMCELL Technologies) for the first 24 hours. Each experiment was performed in triplicate. Where applicable, sFRP2 (10, 30, or 60 ng/ml) was added to the medium after 24 hours and replenished in every medium change. Organoids were processed for OCT embedding for immunostaining or made into a single cell preparation and positively selected for Epcam for RNA extraction.

#### PCLSs.

Fresh lung tissues were obtained from normal donors and from IPF patients that underwent lung transplantation. PCLSs were prepared as described (12). Lung tissues were inflated with warm 2% low-melting agarose (catalog 16550100, Thermo Fisher) and placed in cold PBS. Solidified tissues were cut into 400 μm thick slices using Compresstome (catalog VF-310-0Z; Precisionary Instrument LLC.) One randomly selected slice per well in a 24-well plate was cultured in serum-free DMEM supplemented with 100 units/mL penicillin and streptomycin under standard cell-culture conditions (37°C, 5% CO_2_, 100% humidity) with Nystatin (catalog N186, Thermo Fisher, 20 U/ml^–1^). Where applicable, TGF-β1 (2 ng/ml) and sFRP2 (60 ng/ml), were added to the medium and replenished in every medium change. At day 5 or 7, all cultured PCLSs were immediately transferred into liquid nitrogen and subsequently stored at −80°C prior to protein extraction.

### Immunofluorescence and in situ hybridization

#### Paraffin embedding.

Diagnosis biopsy samples from patients were fixed in 4% paraformaldehyde (PFA) overnight at 4°C. The lungs were then washed with PBS 4 times for 30 minutes each at 4°C, then dehydrated in a series of ethanol solutions (30%, 50%, 70%, 95%, and 100%). The dehydrated lungs were incubated with xylene for 1 hour, then embedded in paraffin. The lungs were sectioned at 8 μm on a microtome.

#### OCT embedding.

Lungs inflated with 94% OCT/2% PFA/4% PBS and organoids in 3D Matrigel were fixed with 4% PFA for 1 hour, washed with PBS 3 times, and embedded in OCT after 30% and 15% sucrose gradient washing. Sections (8 μm) were cut on a cryostat.

#### Immunofluorescent staining.

OCT-embedded slides were fixed in 4% PFA for 10 minutes, then washed with PBS. Antigen retrieval (catalog DV2004MX, Biocare) was performed for 20 minutes at 95°C or at 155°C and the slides were then washed in distilled water. Slides were washed with PBS, blocked/permeabilized (5% horse serum, 0.5% BSA, 0.1% Triton X-100) for 1 hour, and then incubated with primary antibodies overnight at 4°C (Supplemental Table 7). Slides were washed with PBS and then incubated with secondary antibodies for 1 hour (Supplemental Table 7). Prior to mounting, DAPI was added for 5 minutes, and slides were mounted with ProLong Gold (Thermo Fisher Scientific). Images were captured using ZEN, version 3.1, software (Zeiss). Where indicated, multiple images at ×20 were captured using the MosaiX function and stitched together using the Tile Stitch function in ZEN.

#### In situ hybridization.

Sections of 7 μm were made from formalin-fixed, paraffin-embedded tissue blocks and used for RNAscope Fluorescent Multiplex Assay, version 2 (catalog 323110, ACDBio) according to manufacturer protocol. Briefly, protease treatment was performed, followed by hybridization of probes against SFTPC (catalog 452561, ACDBio), COL1A2 (catalog 432721, ACDBio), KRT17 (catalog 463661 ACDBio), and SFRP2 (catalog 476341, ACDBio) mRNAs (Supplemental Table 6).

### Gene expression analysis

#### Single-cell preparation from organoids and AEC2-2D.

The cell-Matrigel mixture in the Transwell was washed with PBS and incubated in 15 U.ml^–1^ dispase for 30–45 minutes at 37°C with intermittent resuspension. The mixture was removed from the Transwell and resuspended in TrypLE (catalog 12563011, Thermo Fisher). Cells were shaken at 37°C for up to 20 minutes, with pipetting up and down 10 times every 5 minutes, checked for single cells, and stained with biotin anti-CD326 (catalog 324216, BioLegend) for 30 minutes at 4°C. Streptavidin beads (catalog 17663, STEMCELL Technologies) were added to isolate the epithelial cells, and the rest of the cells were mesenchymal cells. AEC-2D cells were washed twice with PBS. Dispase (15 U/ml) was added, and plate was incubated for 35 minutes with shaking at 37°C. Dispase was carefully collected from the wells without disturbing the Matrigel. Wells were washed twice with PBS to ensure recovery of all cells.

#### RNA extraction.

RNA was extracted using the ReliaPrep RNA Cell Miniprep System (catalog Z6011, Promega) per the manufacturer’s instructions.

#### qPCR.

Reverse transcription was performed with iScript RT Supermix (catalog 1708841 Bio-Rad), and qPCR was performed using SsoAdvanced Universal SYBR Green Supermix (catalog 1725271, Bio-Rad). Relative expression was calculated with the ΔΔ method. A list of primers is provided in Supplemental Table 5.

### Protein expression

Pulverized PCLS tissues were lysed in RIPA buffer and analyzed by immunoblotting as previously described (12). Densitometry was quantified using ImageJ software (NIH). A list of antibodies is provided in Supplemental Table 7.

### 293 Cell culture and transfection

293 Cells (catalog CRL1573, ATCC) were cultured in DMEM supplemented with penicillin/streptomycin and 10% FBS. All the cell lines in the laboratory are periodically tested for mycoplasma contamination. Only the mycoplasma-free cells are used for experiments. For transfection, 293 cells with 80% confluency were cotransfected with FZD plasmid (GenScript) using TurboFect Transfection Reagent (catalog R0531, Thermo Fisher) and cultured in DMEM complete medium for 24–48 hours. Cells were washed with PBS 3 times and replaced with serum-free medium with or without SFRP2 (30 ng/ml) for 1 hour at 37°C before lysis for nuclei isolation.

### Isolation of nuclei

293 Cells transfected with FZD5 were lysed in ice-cold NP40 lysis buffer (5 mM Tris, pH 8.0, 15 mM NaCl, and 0.1% NP40) supplemented with protease/phosphatase inhibitors and 1 mm phenylmethylsulfonyl fluoride. After 5 minutes incubation with lysis buffer, cells were scraped off and centrifuged at 500 *g* for 5 minutes at 4°C. Supernatant was saved as cytosol control. Nuclei pellet was washed with lysis buffer once before solubilizing in RIPA buffer. Clarified nuclei supernatant along with cytosol control were blotted for NFAT3 (catalog sc8405, Santa Cruz Biotechnology Inc.), FZD5 (catalog MA5-17080, Thermo Fisher), GAPDH (catalog sc-47724, Santa Cruz Biotechnology Inc.), or NUP62 (catalog 13916-AP, Thermo Fisher).

### Image quantification

Sections were imaged for quantification on a Zeiss AxioImager.M1 microscope. Cell counts for stained organoids were performed manually. Approximatively 1,000 cells per condition were counted. The results were averaged between specimens, and SD values were calculated per condition. For RNAscope images, nuclei (DAPI) were used to locate and identify individual cells. To avoid overlapping nuclei, area, circularity, and intensity range were restricted (28.639 to approximately 154.142 μm^2^; 0.644 to approximately 0.883; gray level 41.00 to approximately 1907.00, respectively). Signals (SFTPC, GFP; sFRP2, RFP) that overlapped with nuclei were counted as positive. Coordinates of positive cells were then measured by center of nuclei to *x* axis and *y* axis. Direct distance of sFRP2^+^ cells (#1) to SFTPC^+^ cells (#2) was calculated as follows: √(*X_2_* – *X_1_*)^2^ + (*Y_2_* – *Y_1_*)^2^. For quantification in biopsies, the level of expression of sFRP2 was measured as the highest value in the largest area in the look-up table (LUT).

### Statistics

Statistical analysis of scRNA-Seq gene expression was performed in Seurat with MAST. Overlapping gene lists were compared with those in Nematode Bioinformatics (nemates.org) with a base value of 30,000 genes in the genome. Cell-composition analysis was done using Excel, version 200302, with a 2-tailed *t* test. Statistical analyses for cell count and gene and protein expression were performed using GraphPad Prism. One-way ANOVA and unpaired and paired 2-tailed *t* tests were used to determine *P* values, and the data in graphs are presented as mean ± SD. Unpaired *t* test was used to compare 2 treatment groups. The Kruskal-Wallis test or Dunnett’s multiple-comparisons test was used for multiple comparisons. For normally distributed data, ordinary 1-way ANOVA followed by Tukey’s multiple-comparisons test was performed.

### Study approval

The study protocol for enrollment of ILD patients prior to biopsy into EGCG-treated versus control cohorts was reviewed and approved by the UCSF Institutional Review Board and prospectively entered at ClinicalTrials.gov (NCT03928847). Tissue from normal lungs declined for transplantation and from IPF patients undergoing transplantation were deidentified and donated to research through institutional protocols approved by the UCSF Institutional Review Board. All patients provided written, informed consent.

### Data availability

RNA-Seq data reported in this paper have been deposited in the NCBI’s Gene Expression Omnibus (GEO GSE239664). Previously published scRNA-Seq data that were included in the analysis included GEO GSE135893 and GSE132771. Publicly available R packages were used for all computational analyses. No custom codes were developed. Representative code is available on request. Values for all data points in graphs are reported in the Supporting Data Values file. All raw data used for quantification and statistical analysis are available in a single GraphPad Prism file that is available upon request.

## Author contributions

MLC, YW, HAC, and CJLS conceived the study. ANB, TCH, YW, KG, MLC, and CJLS performed the experiments. MLC analyzed scRNA transcriptome data. ANB, HAC, TCH, and CJLS analyzed cell biology data. YW analyzed biochemical data. MLC, GM, DL, JAG, YW, and HAC recruited ILD patients, and BNT and DMJ performed surgical lung biopsies. MAM, KDJ, and PJW provided human lung samples. DL and YW coordinated surgical lung tissue collection. VWD recruited donors. MLC, HAC, and CJLS wrote the manuscript. All authors read and reviewed the manuscript. MLC and ANB were designated co–first authors based on their relative overall contributions to performing and analyzing the bioinformatic and cell biology aspects of the study, respectively. MLC was listed first for his input to the conception of the study.

## Supplementary Material

Supplemental data

Unedited blot and gel images

Supplemental table 2

Supplemental table 3

Supporting data values

## Version 1 07/09/2024

In-Press Preview

## Version 2 09/17/2024

Electronic publication
