# Supplementary material for: A fibroblast-dependent TGF-β1/sFRP2 noncanonical Wnt signaling axis promotes epithelial metaplasia in idiopathic pulmonary fibrosis
Source: J Clin Invest. 2024 Jul 9;134(18):e174598. doi: 10.1172/JCI174598 (PMC11405054; doi:10.1172/JCI174598)
Supplement: Supplemental data [file jci-134-174598-s221.pdf]

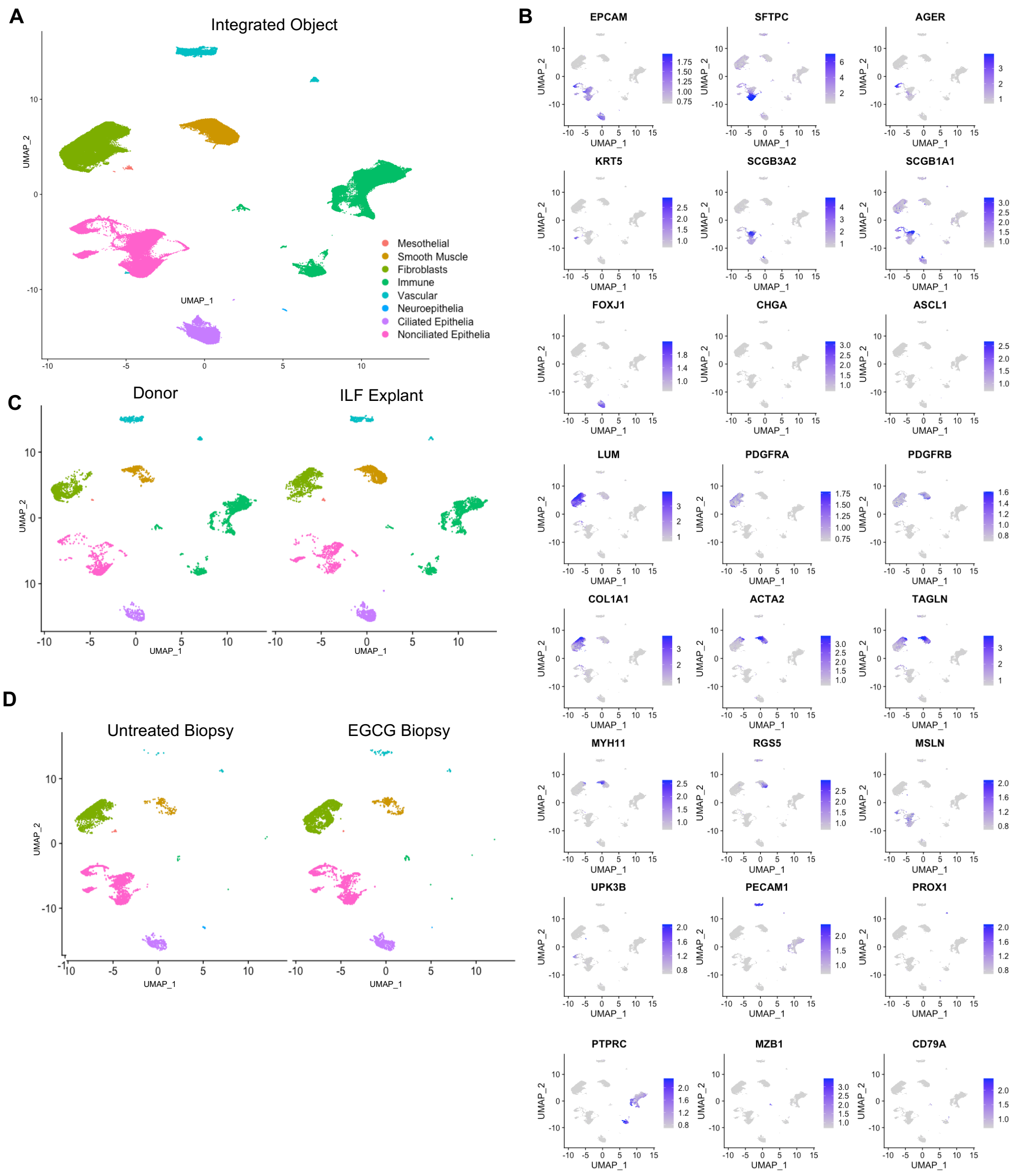

Supplemental Figure 1: (A) UMAP dimension plot, following integration of 48 different scRNA samples into a single Seurat object containing 232,513 cells, showing major annotated cell types, including mesothelial (754 cells), smooth muscle (27,596 cells), fibroblasts (55,242 cells),

immune (62,956 cells), vascular (13,167 cells), neuroepithelia (636 cells), ciliated epithelia (21,474 cells), and non-ciliated epithelia (50,688). **(B)** Cell type identification was based on canonical genes, with selected genes shown here. For example, Nonciliated epithelia express EPCAM, SFTPC, AGER, KRT5, SCGB3A2, SCGB1A1; Ciliated epithelia express EPCAM, FOXJ1; Neuroendocrine cells express CHGA and ASCL1; Fibroblasts express LUM, PDGFRA, COL1A1; Smooth Muscle express PDGFRB, ACTA2, TAGLN, MYH11, RGS5; Mesothelia express MSLN, UPK3B; Vascular Cells express PECAM1 and PROX1; and Immune Cells express PTPRC, MZB1, CD79A. **(C, D)** UMAP dimension plot with 5,000 randomly selected cells per sample type, with sample cell type annotation coloring as in (A).

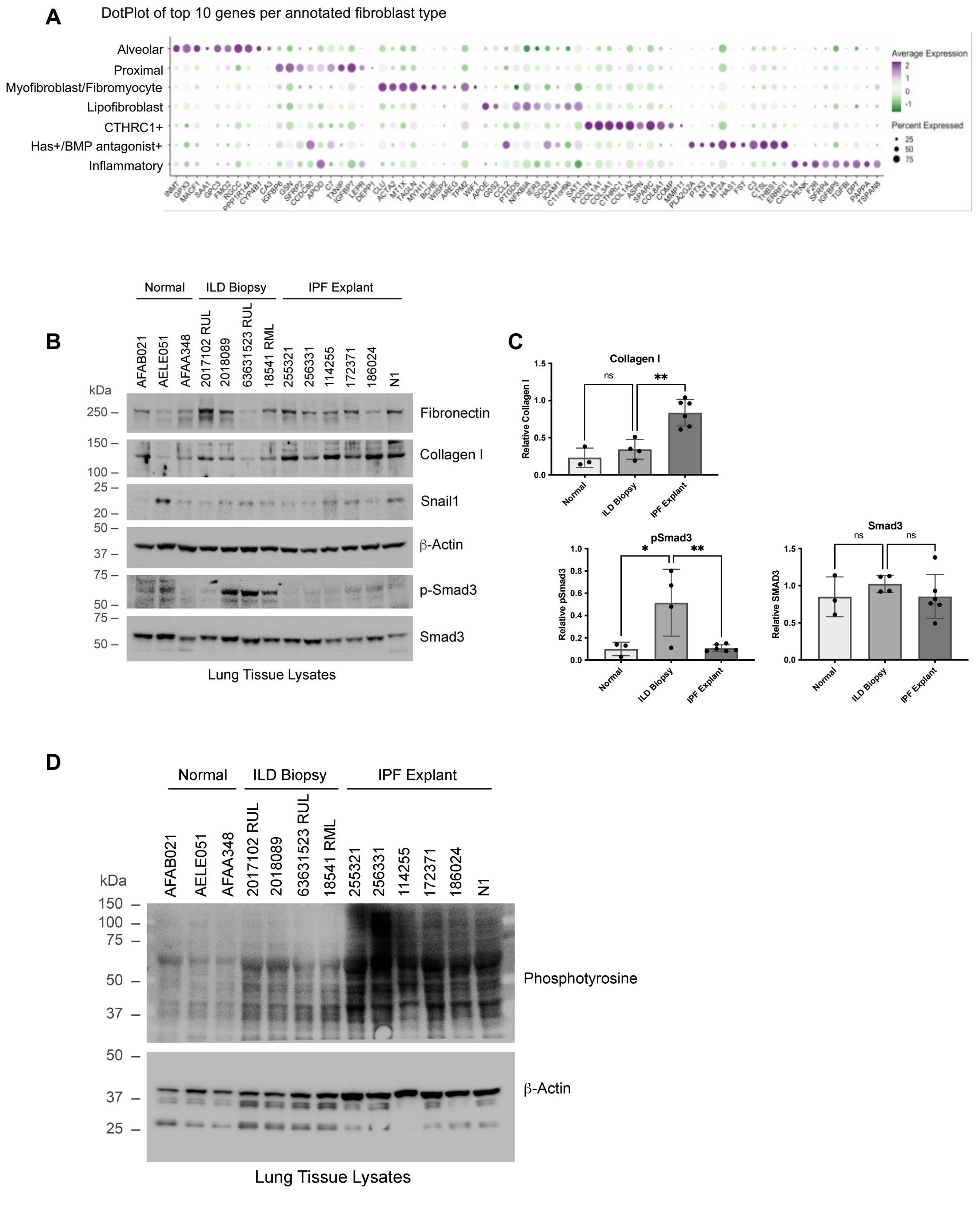

Supplemental Figure 2. (A) Dot Plot of top subtype-specific genes in fibroblast subtypes. **(B-C)** Western Blot of lysates from donor (n= 3), untreated biopsy (n=4), and IPF explants (n=6) showing highest pSmad3 expression in untreated biopsies. **(C)** Quantification of collagen I,

pSmad3, and Smad3 expression. **(D)** Western Blot of lysates from donor, control biopsy, and explant lungs with a pan-phosphotyrosine antibody. Statistical significance was determined by One-way ANOVA followed by Dunnett’s test.

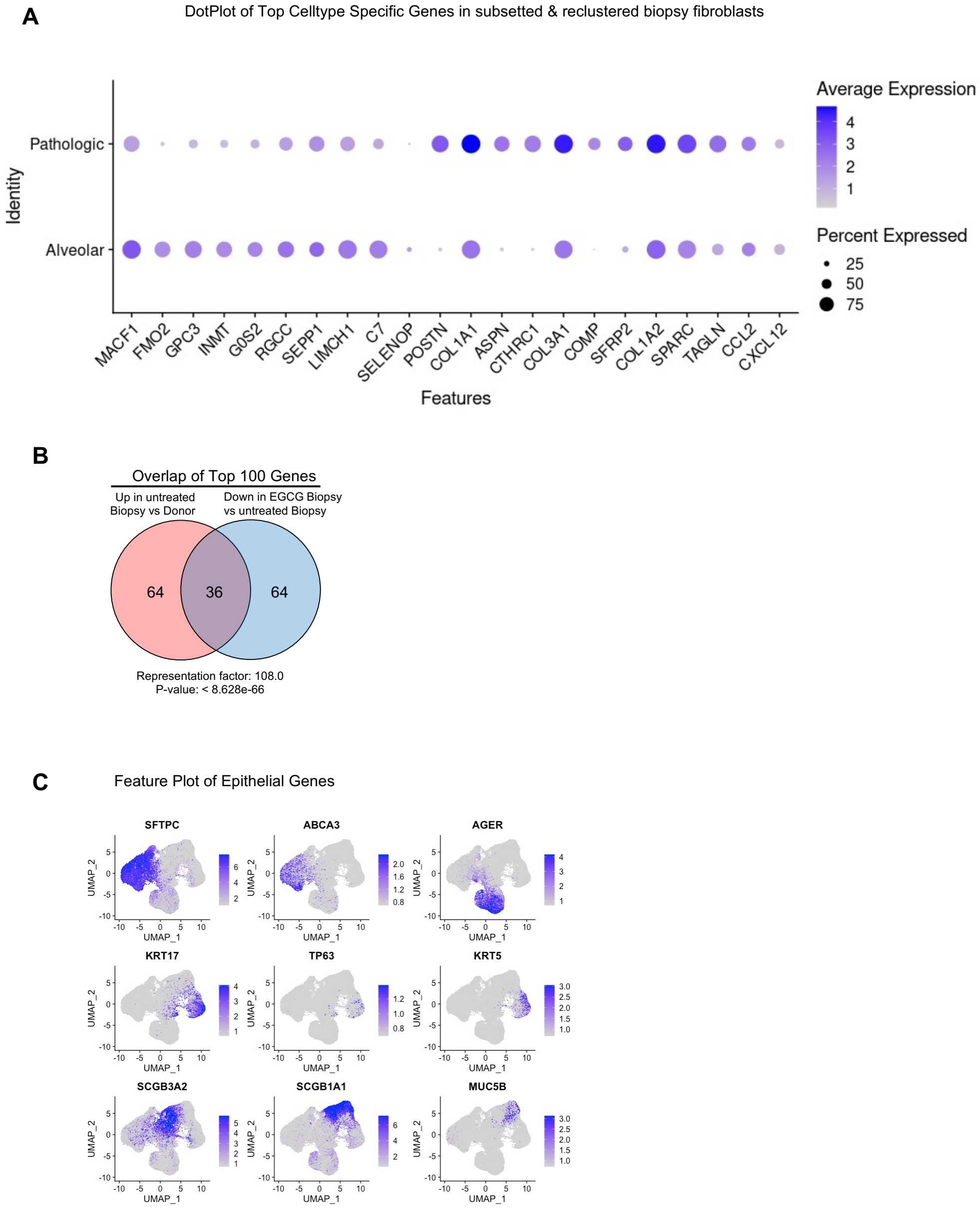

Supplemental Figure 3. (A) Feature Plot of selected fibrosis-related genes in subsetted & reclustered fibroblasts from untreaed biopsy and EGCG biopsy. **(B)** Overlap between the 100 most upregulated genes in the untreated biopsy vs non-diseased donor D.E. list and the 100 most downregulated genes in the EGCG biopsy vs untreated biopsy D.E. list. Statistical analysis indicates the overlap is highly significant (non-random), confirming a role for TGFβ1 signaling in the disease process. **(C)** Feature Plot of epithelial cell type specific genes, used for annotation in Figure 3E.

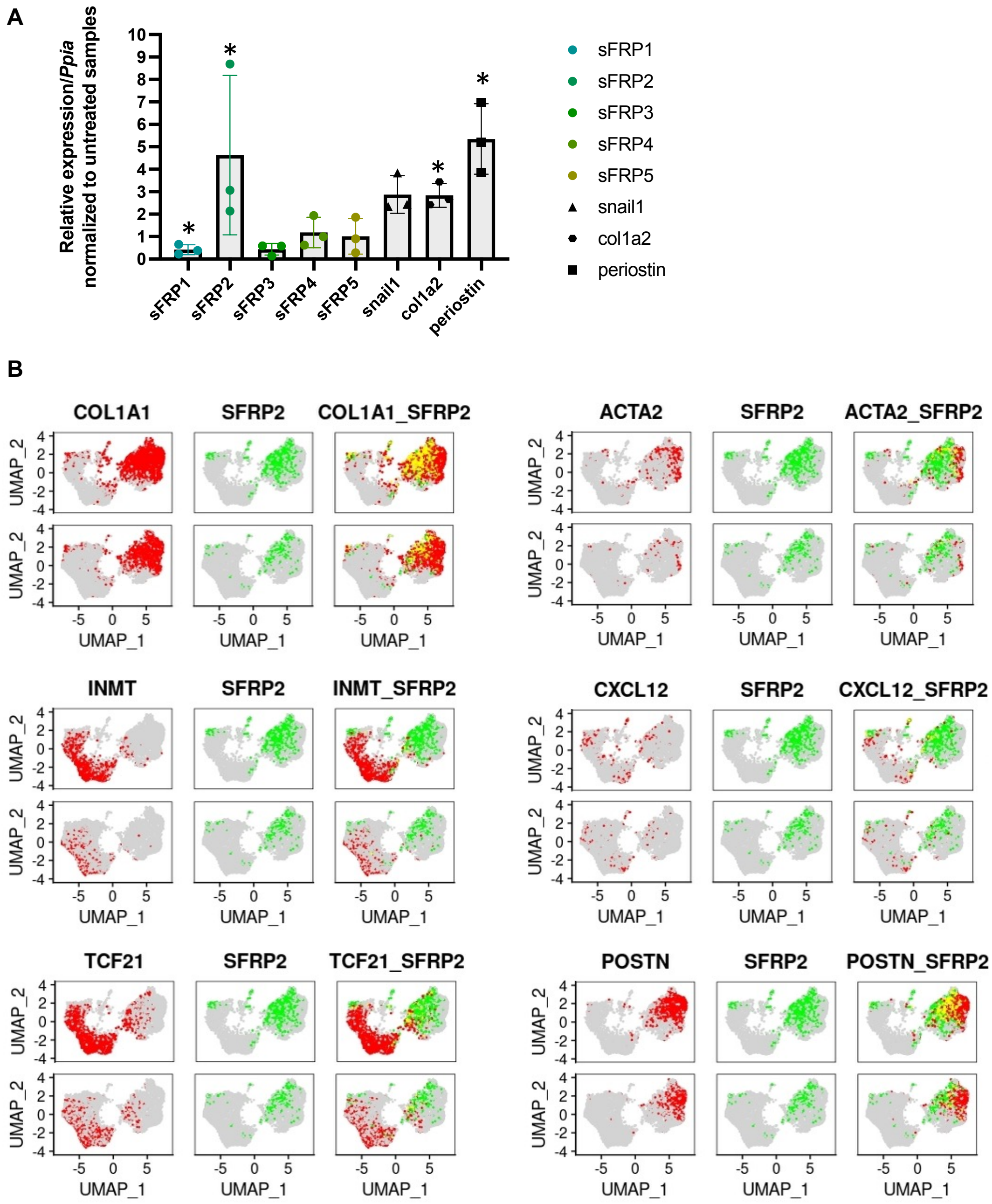

Supplemental Figure 4. (A) Relative RNA expression of five members of the sFRP family (*sfrp1*, *sfrp2*, *sfrp3*, *sfrp4*, and *sfrp5*) and known TGF-β1 target genes (*snail1*, *col1a2*, *periostin*) in human fibroblasts treated with TGFβ1 (1 ng.ml^{-1}) for 48 h (n=3). Statistical significance was determined by 2-tailed t-test. **(B)** Feature plots of *sfrp2* gene expression (green) in various fibroblast subsets characterized by *col1a1, acta2, inmt, cxcl12 ,tcf21, and periostin (postn)* gene expression (red). Cells expressing both *sfrp2* and marker gene are indicated in yellow.

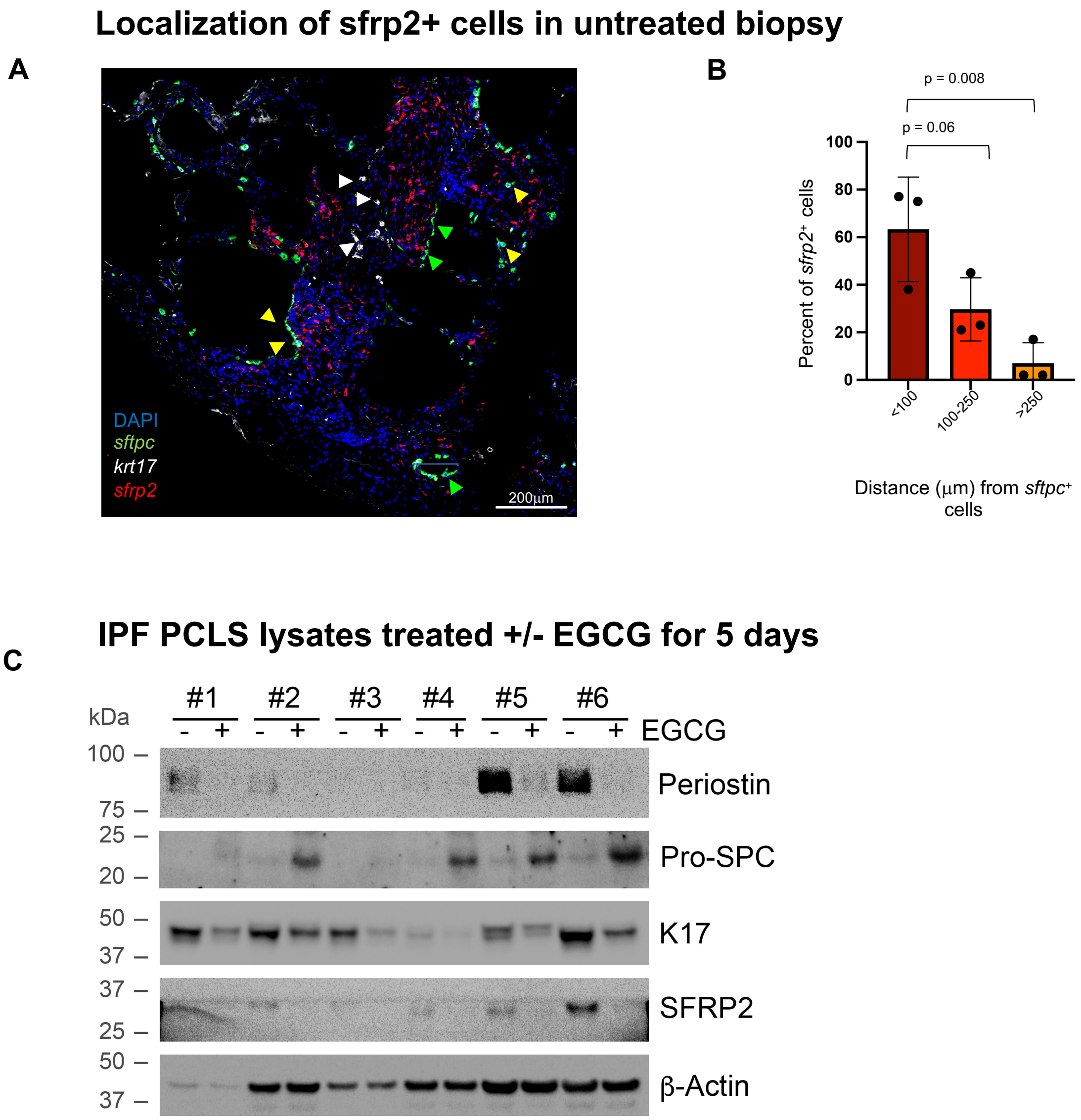

Supplemental Figure 5. (A) RNA *in situ* hybridization for *sftpc, krt17* and *sfrp2* on an IPF biopsy sample, with green arrowheads indicating *sftpc*$^+$/*krt17*$^-$ cells, yellow arrowheads indicating *sftpc*$^+$/*krt17*$^+$ cells, and white arrowheads indicating *sftpc*$^-$/*krt17*$^+$ cells. Representative image of n=3 samples. Magnification x100. **(B)** Quantification of proximity of *sfrp2*$^+$ cells to *sftpc*$^+$ cells. Statistical significance was determined by One-way ANOVA tests followed by Dunnett's multiple comparisons test. Full panel for Western Blot analysis of lysates from precision-cut lung slices from IPF lung donors cultured for 7 days with EGCG (1mM).

A

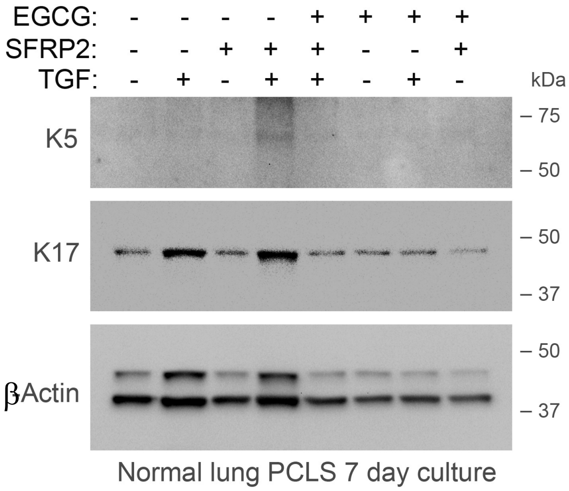

B

Quantification for the expression of Krt 5 and Krt 17 in PCLS treated with TGF-β1/sFRP2/EGCG

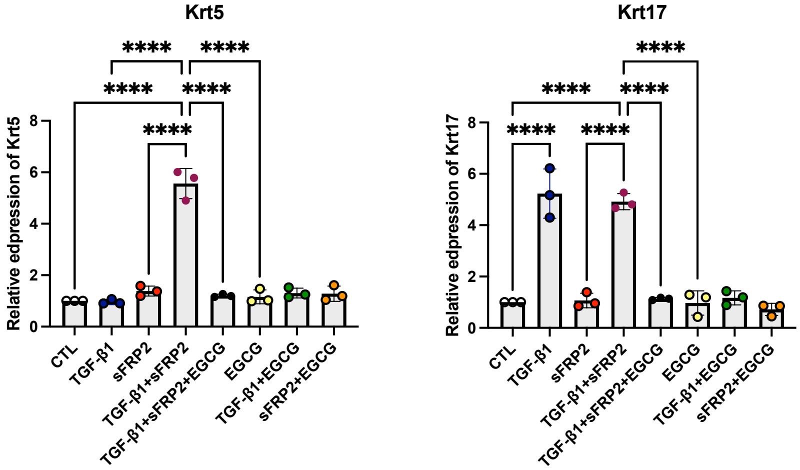

Supplemental Figure 6. (A) Full panel for Western Blot analysis of lysates from precision-cut lung slices from non-diseased lung donors cultured for 7 days with +/- TGFβ1 (2 ng.ml^{-1}) +/- sFRP2 (60 ng.ml^{-1}) +/- EGCG (1uM) for 7 days. Lysates were blotted for KRT5 and KRT17. n= 3 biological replicates. (B) Graphical representation of the level of expression of KRT5 and KRT17. Statistical significance was determined by One-way ANOVA tests followed by Dunnett's multiple comparisons test.

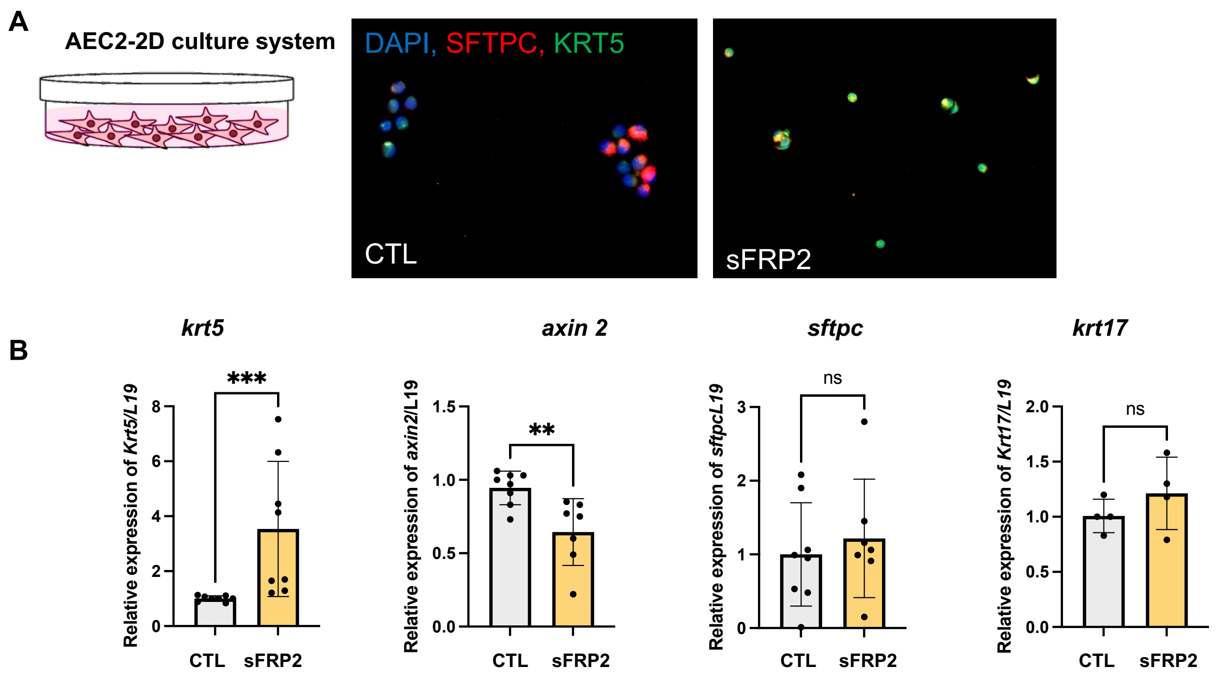

Supplemental Figure 7. (**A**) Immunofluorescence for SFTPC and KRT5 of AEC2-2D culture treated with sFRP2 (60 ng.ml^{-1}). n=3 biological replicates. (B) Level of expression of *krt5*, *axin2, krt17*, and *sftpc* mRNA level in Epcam+ cells isolated from 3-day culture of AEC2s-2D culture treated with sFRP2 (60 ng.ml^{-1}). at least 7 biological replicates. Statistical significance was determined by Mann Whitney t-test.

Supplemental Tables

Biopsy Patient Characteristics

	Biopsy EGCG	Biopsy Control
	n = 4	n=4
**Male sex, No. (%)**	0 (0)	2 (50)
**Age, Mean (SD), years**	61.5 (5.5)	62.8 (6.6)
**FEV1**		
Mean (SD), L	2.18 (0.37)	2.78 (1.26)
Median (IQR), L	2.14 (1.89-2.44)	2.40 (1.95-3.23)
% Predicted value, mean (SD)	80.7 (12.4)	88.8 (13.9)
**FVC**		
Mean (SD), L	2.69 (0.59)	3.44 (1.40)
Median (IQR), L	2.67 (2.23-3.13)	3.04 (2.53-3.95)
% Predicted value, mean (SD)	76.8 (15.4)	82.3 (11.1)
**FEV1/FVC, Mean (SD), %**	81.9	79.7
**TLC**		
Mean (SD), L	4.20 (1.26)	4.97 (1.31)
Median (IQR), L	3.78 (3.40-4.57)	4.46 (4.17-5.26)
% Predicted value, mean (SD)	77.0 (21.4)	82.0 (3.9)
**Hgb-corrected Dlco, % predicted value, mean (SD)**	65.0 (21.1)	66.5 (20.6)
**Patient**	**Diagnosis**	**Biopsy # (scRNA metadata)**
Control Biopsy 1	Unclassifiable	Biopsy 1
Control Biopsy 2	UIP	Biopsy 2
Control Biopsy 3	NSIP	Biopsy 6
Control Biopsy 4	UIP	Biopsy 7
EGCG Biopsy 1	UIP + Nonspecific	Biopsy 3
EGCG Biopsy 2	cHP	Biopsy 4
EGCG Biopsy 3	UIP	Biopsy 5
EGCG Biopsy 4	UIP	Biopsy 8

Supplemental Table 1: Characteristics of patients who donated biopsy tissue, untreated and EGCG-treated.

Supplemental Table 2 and Supplemental Table 3 have been uploaded as Supplemental Data (exel files)

Supplemental Table 4: Statistical significance related to Figure 5

4A: Statistical significance related to Figure 5B

Tukey's multiple comparisons tes	Adjusted P Value
SFTPC+/Krt5-	
CTL vs. sFRP2 10	0.9952
CTL vs. sFRP2 30	0.0069
CTL vs. sFRP2 60	<0.0001
sFRP2 10 vs. sFRP2 30	0.0101
sFRP2 10 vs. sFRP2 60	<0.0001
sFRP2 30 vs. sFRP2 60	0.0526
SFTPC-/Krt5+	
CTL vs. sFRP2 10	
CTL vs. sFRP2 30	0.6919
CTL vs. sFRP2 60	<0.0001
sFRP2 10 vs. sFRP2 30	0.8114
sFRP2 10 vs. sFRP2 60	<0.0001
sFRP2 30 vs. sFRP2 60	0.0004
SFTPC+/Krt5+	
CTL vs. sFRP2 10	0.9956
CTL vs. sFRP2 30	0.0227
CTL vs. sFRP2 60	0.501
sFRP2 10 vs. sFRP2 30	0.0457
sFRP2 10 vs. sFRP2 60	0.8876
sFRP2 30 vs. sFRP2 60	0.0162

4B: Statistical significance related to Figure 5D

Šídák's multiple comparisons test	Adjusted P Value
CTL - si sFRP2	
SFTPC+/Krt5-	0.0303
SFTPC+/Krt5+	0.001
SFTPC-/Krt5+	0.226

Supplemental Table 5: List of primers used for qPCR

Target gene	Forward	Reverse
Human sFRP2	CGA GGA AGC TCC AAA GGT ATG TGA	TCG GAC ACA CCG TTC AGC T
Human Krt5	GGA ATG CAG ACT CAG TGG AGA	GCT GCT GGA GTA GTA GCT TCC
Human Axin2	TAC ACT CCT TAT TGG GCG ATC A	TTG GCT ACT CGT AAA GTT TTG GT
Human Frizzled 5	TGG AAC GCT TCC GCT ATC CTG A	GGT CTC GTA GTG GAT GTG GTT G
Human Frizzled6	GGC AGT GTA TCT GAA AGT GCG C	GAT GTG GAA CCT TTG AGG CTG C
L19	ATG TAT CAC AGC CTG TAC CTG	TTC TTG GTC TCT TCC TCC TTG
PPIA	GCA TAC GGG TCC TGG CAT CT	ACT TTG CCA AAC ACC ACA TGC T
Human Periostin	CGG GAC CAA GGC CCA AAT GT	GCT GGG CAG CCT TTC ATT CTT
Human Snail	CTG AGG CCA AGG ATC TCC AG	GAC AGG AGA AGG GCT TCT CG
Human Collagen 1A2	GTT GCT GCT TGC AGT AAC CTT	AGG GCC AAG TCC AAC TCC TT
Human p63	GCC ATG CCC AGT ATG TAG AAG	TCA TCC CTC CAA CAC AAC TG
Human NGFR	CCT CAT CCC TGT CTA TTG CTC C	GTT GGC TCC TTG CTT GTT CTG C
Human Krt19	AGC TAG AGG TGA AGA TCC GCG A	GCA GGA CAA TCC TGG AGT TCT C
Human Krt17	GGT GGG TGG TGA GAT CAA TGT	CGC GGT TCA GTT CCT CTG TC
Human SFRP1	ATG CCA CCG AAG CCT CCA A	GCA AAC TCG CTG GCA CAG A
Human SFRP2	CGA GGA AGC TCC AAA GGT ATG TGA	TCG GAC ACA CCG TTC AGC T
Human SFRP3	GCT ACA CAG AAG ACC TAT TTC CG	CCG TGG AAT GTT TAC CAG AGA GG
Human SFRP4	GTG GCG CTC AAG GAT GAT GCT	AAC TGT TCT CCG CTG TTC CTG CA
Human SFRP5	TGC TGC ACT GCC ACA AGT T	GTG CTC CAT CTC ACA CTG GG
Human PCKzeta	GTT CTC CTG GTG CGG TTG AAG A	GGT TGC TGG ATG CCT GCT CAA A

Supplemental Table 6: List of probes used for RNAscope and silencing gene expression

Name of probe	Catalog Number	Dilution	Company
RNAscope™ Probe- Hs-SFRP2	476341	N/A	ACD Bio
RNAscope™ Probe- Hs-SFTPC	452561	1:50	ACD Bio
RNAscope™ Probe- Hs-KRT17	463661	1:50	ACD Bio
RNAscope™ Probe- Hs-COL1A2	432721	1:50	ACD Bio
Silencer Select Negative control	4390846		Ambion
Silencer Select sFRP2	4392420 s12718		Ambion
Silencer Select Frizzled-5	4390824 s15416		Ambion
Silencer Select Frizzled-6	s729		Ambion

Supplemental Table 7: List of antibodies used for cell sorting FACS, western blot, and immunofluorescence.

Antibodies used for FACS analysis

Name	Catalog number	Company	Dilution
live dead draq7	7406	Cell Signaling	
CD45	557833	BD Biosciences	1:200
CD31	563653	BD Biosciences	1:200
CD11b	557754	BD Biosciences	1:200
Epcam CD326	324206	BD Biosciences	1:200
Ht-280	TB-27AHT2-280	Terrace Biotech	1:100
Alexa Fluor 488 goat anti IGM	A21042	Invitrogen	1:1000

Antibodies used for Immunofluorescence

Name	Catalog number	Company	Dilution
Anti-Prosurfactant Protein C (proSP-C) Antibody	AB3786	Millipore	1:2500
Cytokeratin 17 (E-4)	sc393002	Santa Cruz Biotechnology	1 to 500
Keratin 5 (Poly9059)	95904	Biolegend	1 to 500

Antibodies used for Western Blot

Name	Catalog number	Company	Dilution
KRT5	MA5-15347	Fisher Scientific	1:1000
KRT17	sc-39300	Santa Cruz Biotechnology	1:100
Periostin	sc-398631	Santa Cruz Biotechnology	1:100
Pro-SFTPC	AB3786	Millipore Sigma	1:1000
sFRP2	MA5-79985	Fisher Scientific	1:1000
b-Actin	A5441	Sigma-Aldrich	1:5000
FZD5	MA5-17080	ThermoFisher	1:500
NFATC3	sc-8405	Santa Cruz Biotechnology	1:200
GAPDH	sc-47724	Santa Cruz Biotechnology	1:200
NUP62	13916-1-AP	ThermoFisher	1:1000
Fibronectin	F3648	Sigma Aldrich	1:500
Snail1	3895	Cell Signaling	1:1000
Smad3			1:1000
pSmad3			1:1000
Phosphotyrosine			
