# Supplementary material for: A fibroblast-dependent TGF-β1/sFRP2 noncanonical Wnt signaling axis promotes epithelial metaplasia in idiopathic pulmonary fibrosis
Source: J Clin Invest. 2024 Jul 9;134(18):e174598. doi: 10.1172/JCI174598 (PMC11405054; doi:10.1172/JCI174598)

Full unedited gel Fig 4F
Anti-Periostin Antibody
Anti-Pro-Spc Antibody
Anti-K17 Antibody
Anti-sFRP2 Antibody
Anti-bActin Antibody

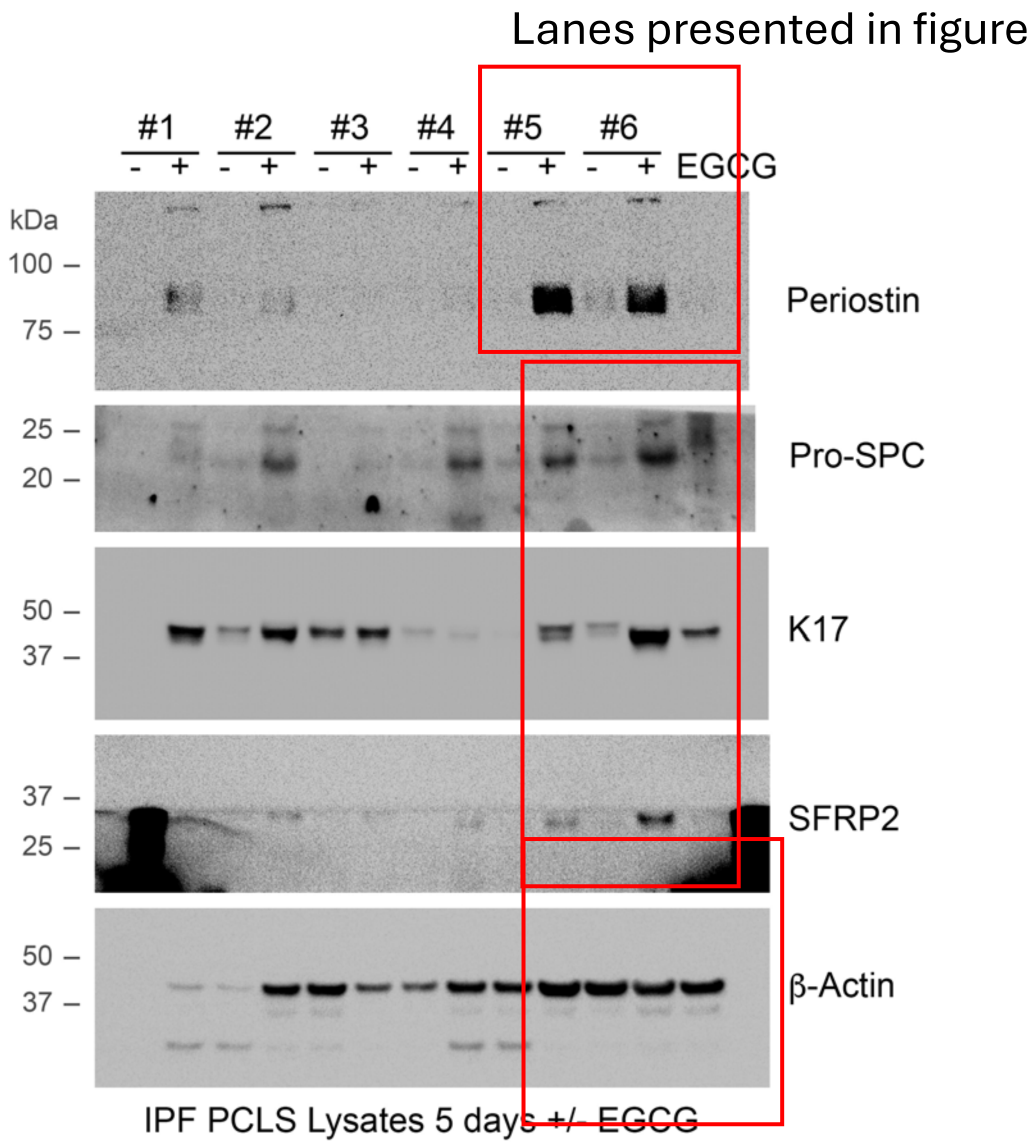

Full unedited gel for Supplem Fig 5C
Anti-Periostin Antibody
Anti-Pro-Spc Antibody
Anti-K17 Antibody
Anti-sFRP2 Antibody
Anti-bActin Antibody

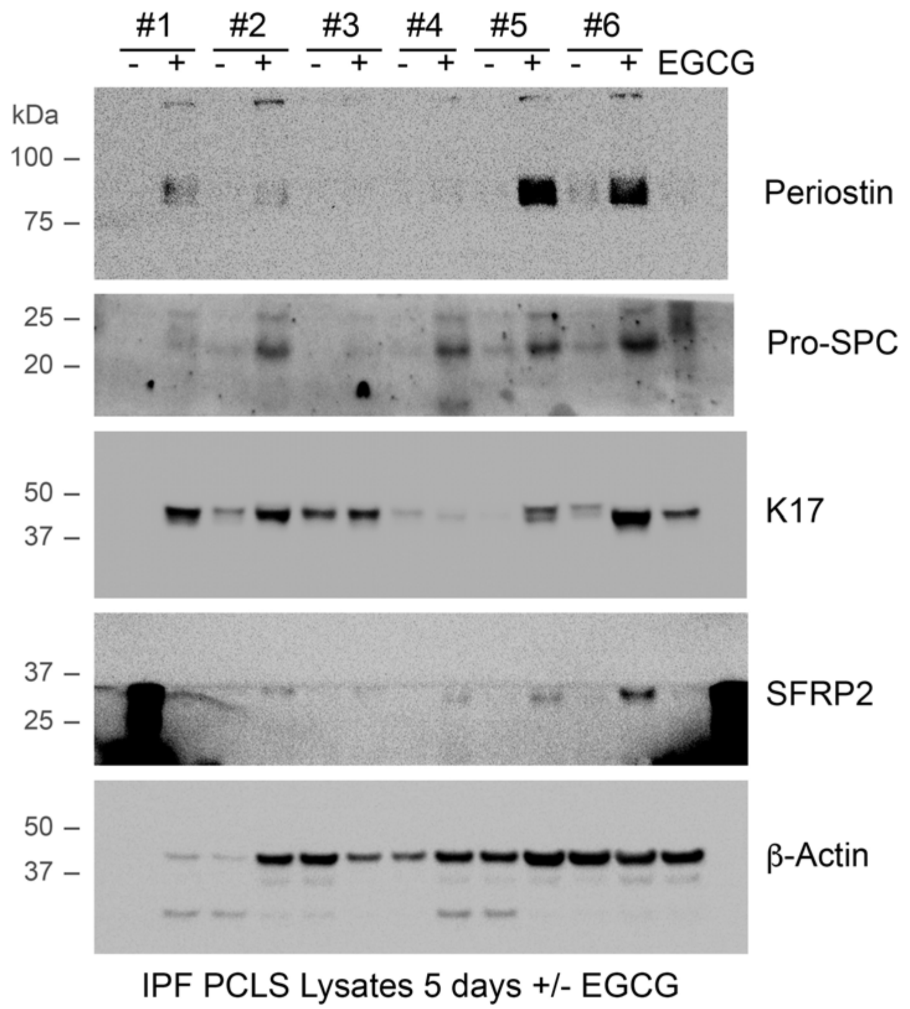

Full unedited gel for Fig 5G
Anti-Keratin 5 antibody
Anti-Keratin 17 antibody
Anti-bActin antibody

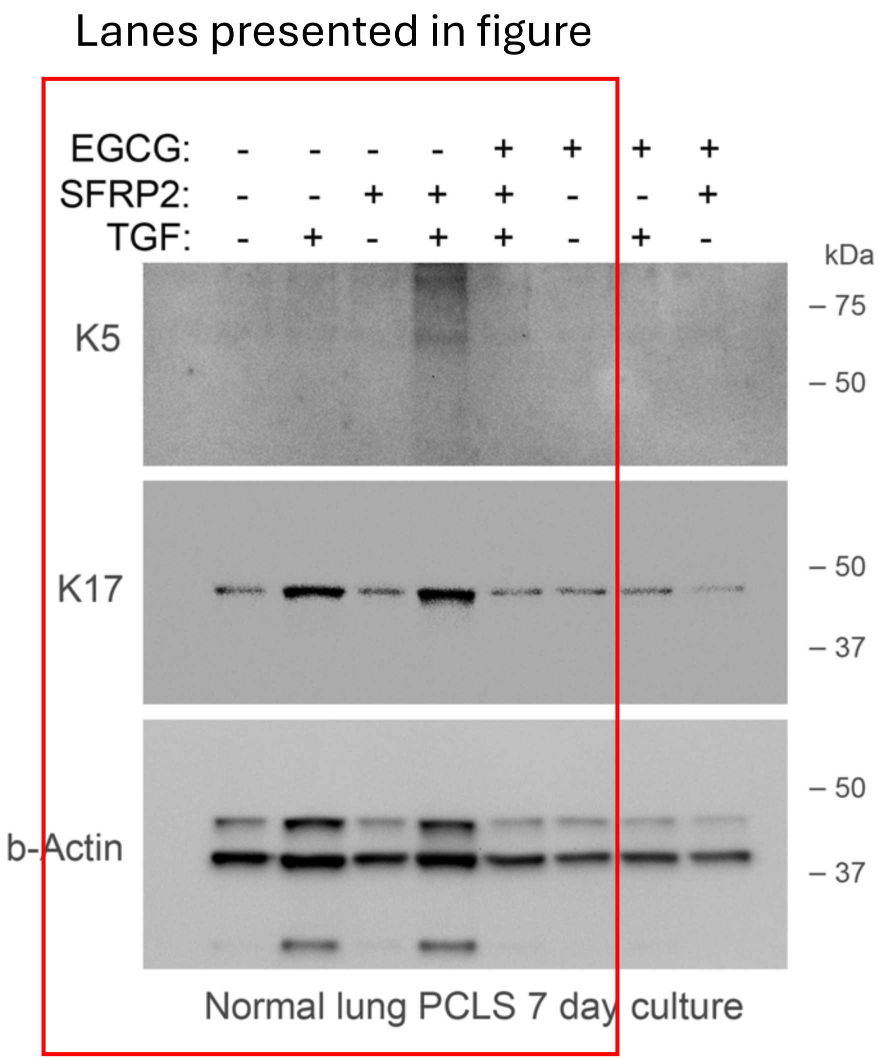

Full unedited gel for Supplem Fig 6A

Anti-Keratin 5 antibody

Anti-Keratin 17 antibody

Anti-bActin antibody

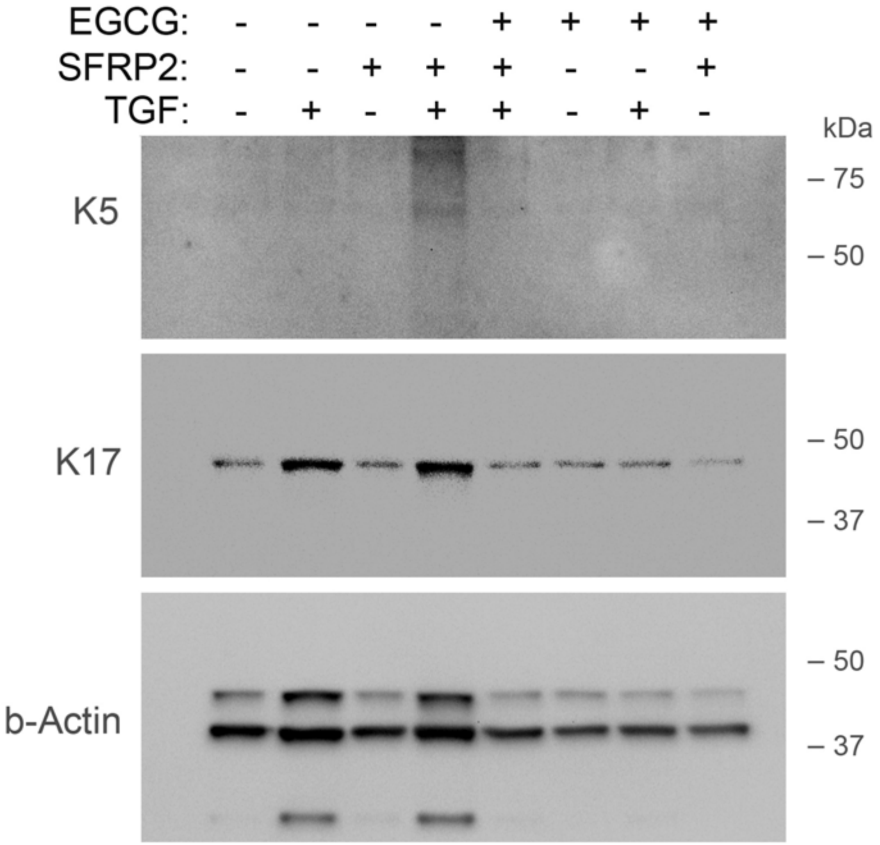

Normal lung PCLS 7 day culture

Full unedited gel for Fig 6F
Anti-NFATC3 antibody
Anti-FZD5 antibody
Anti-GAPDH antibody
Anti-NUP62

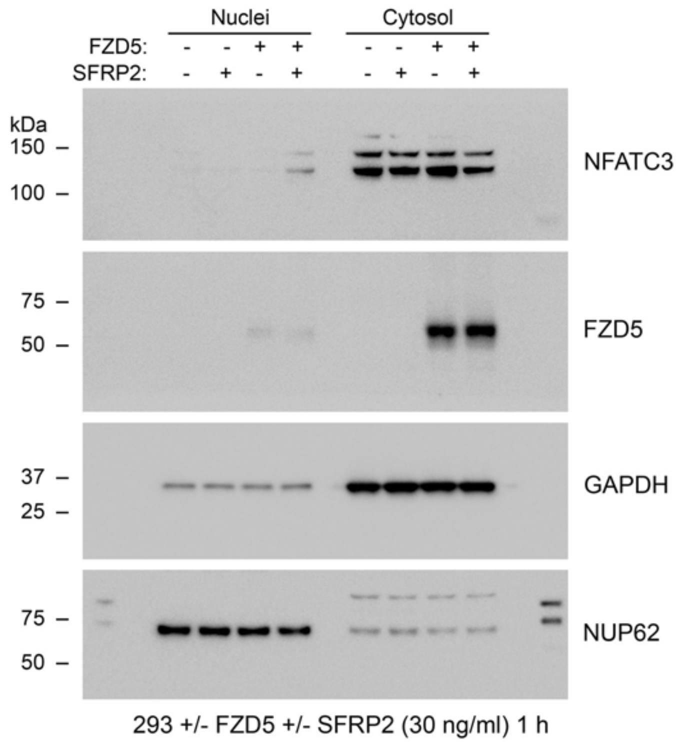

Blot#1

Blot presented in Fig 5F

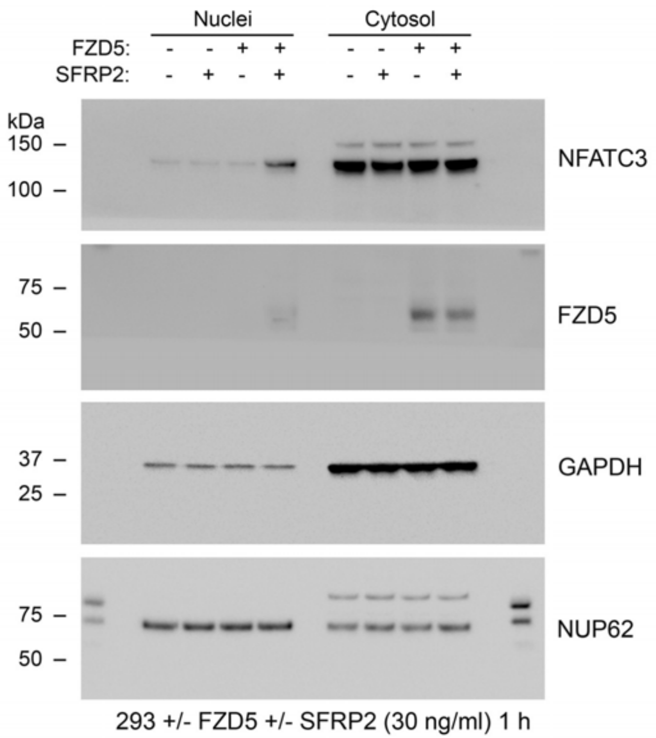

Blot#2

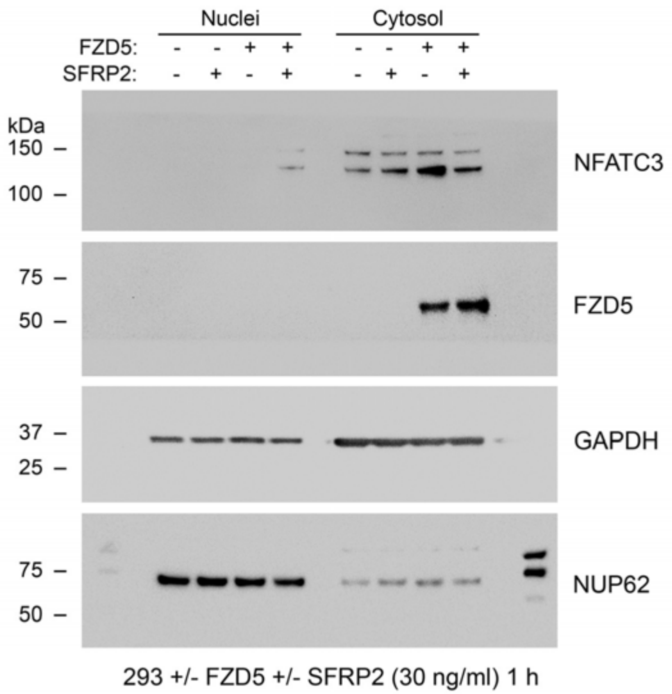

Blot#3

Full unedited gel for Supplem Fig 1B
Anti-Fibronectin antibody
Anti-Collagen I antibody
Anti-Snail antibody
Anti-bActin antibody
Anti-pSmad3 antibody
Anti-Smad3 antibody

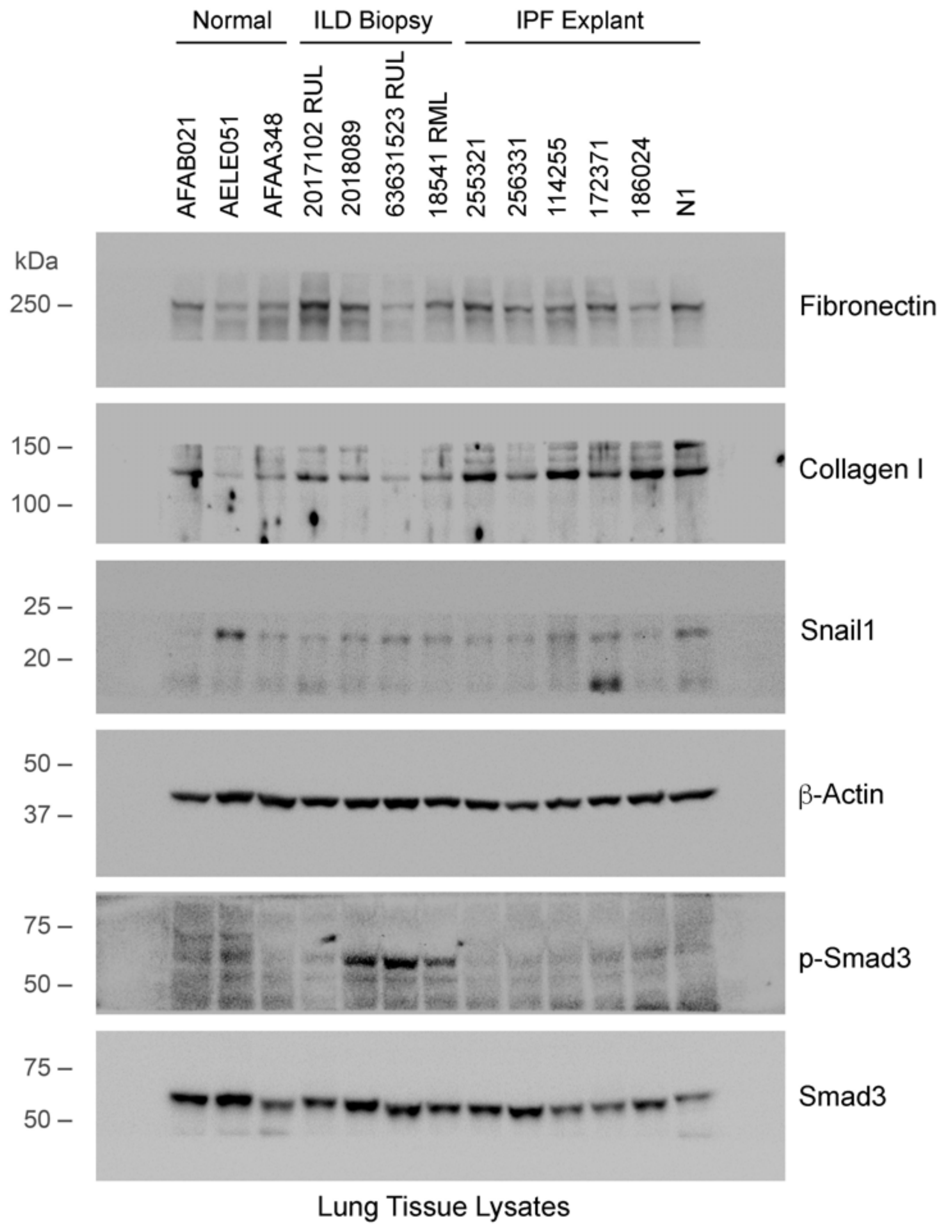

Full unedited gel for Supplem Fig 1D
pan-phosphotyrosine antibody

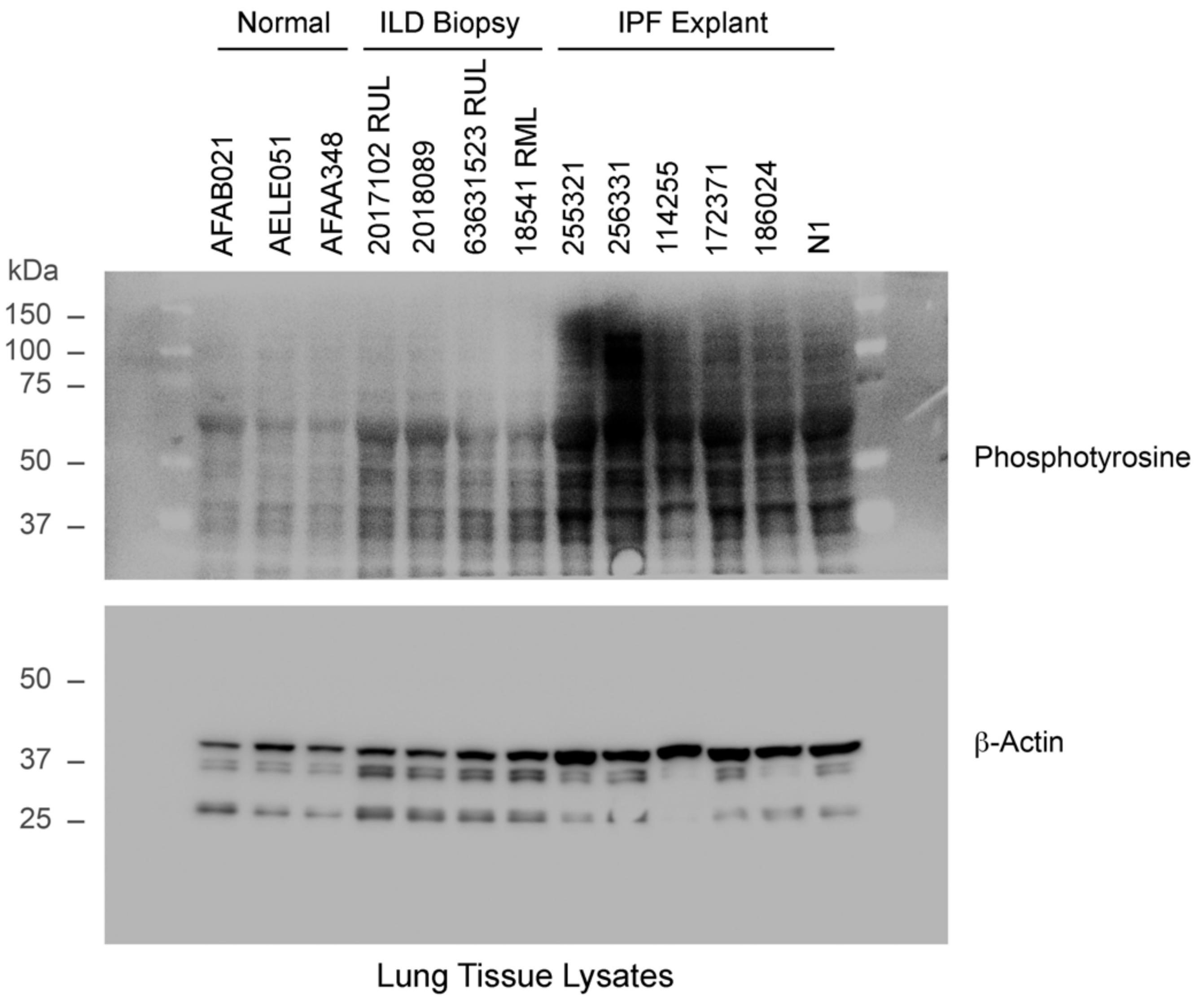

Supplement: Unedited blot and gel images [file jci-134-174598-s222.pdf]
